# Plant *N*-glycan breakdown by human gut *Bacteroides*

**DOI:** 10.1073/pnas.2208168119

**Published:** 2022-09-19

**Authors:** Lucy I. Crouch, Paulina A. Urbanowicz, Arnaud Baslé, Zhi-Peng Cai, Li Liu, Josef Voglmeir, Javier M. Melo Diaz, Samuel T. Benedict, Daniel I. R. Spencer, David N. Bolam

**Affiliations:** ^a^Institute of Microbiology and Infection, College of Medical and Dental Sciences, University of Birmingham, Birmingham B15 2TT, United Kingdom;; ^b^Ludger Ltd, Culham Science Centre, Oxfordshire OX14 3EB, United Kingdom;; ^c^Biosciences Institute, Newcastle University, Newcastle upon Tyne NE2 4HH, United Kingdom;; ^d^Glycomics and Glycan Bioengineering Research Center, College of Food and Technology, Nanjing Agricultural University, 210095 Nanjing, China;; ^e^Chemistry Department, Royal College of Surgeons in Ireland, Dublin 2, Ireland;; ^f^School of Biosciences, University of Birmingham, Edgbaston, University of Birmingham, Birmingham B15 2TT, United Kingdom

**Keywords:** glycobiology, microbiota, plant complex *N*-glycans, glycoside hydrolase

## Abstract

The major nutrients available to the human colonic microbiota are complex glycans derived from the diet. To degrade this highly variable mix of sugar structures, gut microbes have acquired a huge array of different carbohydrate-active enzymes (CAZymes), predominantly glycoside hydrolases, many of which have specificities that can be exploited for a range of different applications. Plant *N*-glycans are prevalent on proteins produced by plants and thus components of the diet, but the breakdown of these complex molecules by the gut microbiota has not been explored. Plant *N*-glycans are also well characterized allergens in pollen and some plant-based foods, and when plants are used in heterologous protein production for medical applications, the *N*-glycans present can pose a risk to therapeutic function and stability. Here we use a novel genome association approach for enzyme discovery to identify a breakdown pathway for plant complex *N*-glycans encoded by a gut *Bacteroides* species and biochemically characterize five CAZymes involved, including structures of the PNGase and GH92 α-mannosidase. These enzymes provide a toolbox for the modification of plant *N*-glycans for a range of potential applications. Furthermore, the keystone PNGase also has activity against insect-type *N*-glycans, which we discuss from the perspective of insects as a nutrient source.

## Significance

*N*-glycans are common posttranslational modifications on plant proteins, particularly secreted proteins. As plants are the major component of the human diet, and especially in high-fiber diets, plant *N*-glycans are prominent in the gut. Despite their ubiquity in the gut, the degradation of plant *N*-glycans by the microbiota has not been described. Here we used a functional analysis approach, coupled to detailed biochemistry and structural biology, to reveal a pathway for the degradation of plant *N*-glycans encoded by the human gut microbiota. The work reveals insight into how our gut microbes use plant *N*-glycans as a nutrient source and also provides tools to modify plant *N*-glycans to mitigate allergic responses, either from foods or plant-expressed therapeutics.

Complex carbohydrates from a wide range of sources are the major nutrients available to the colonic microbiota. Degradation of these complex macromolecules by the microbiota is achieved through expression of a vast array of carbohydrate-active enzymes (CAZymes), with some species of gut microbe encoding >300 CAZyme genes from different families. *Bacteroides* species are particularly adept at glycan breakdown and typically organize the genes encoding the apparatus required for the breakdown of a particular glycan into discrete coregulated loci (polysaccharide utilization loci [PULs]). A typical *Bacteroides* PUL comprises genes encoding CAZymes, the outer membrane glycan import system (SusC/D homologs), surface glycan-binding proteins (SGBPs), and sensor-regulators (1). Discrete CAZyme gene clusters can also exist without the Sus or other PUL components, coregulated with the core PUL apparatus located elsewhere on the genome. As a general rule, the more complex the substrate, the higher the number of CAZymes, CAZy families, and number of loci involved. For example, in *Bacteroides thetaiotaomicron*, the chemically simple substrate starch requires only one PUL, whereas breakdown of highly variable *O*-glycans induces the up-regulation of ∼15 PULs (2).

**Fig. 1.**
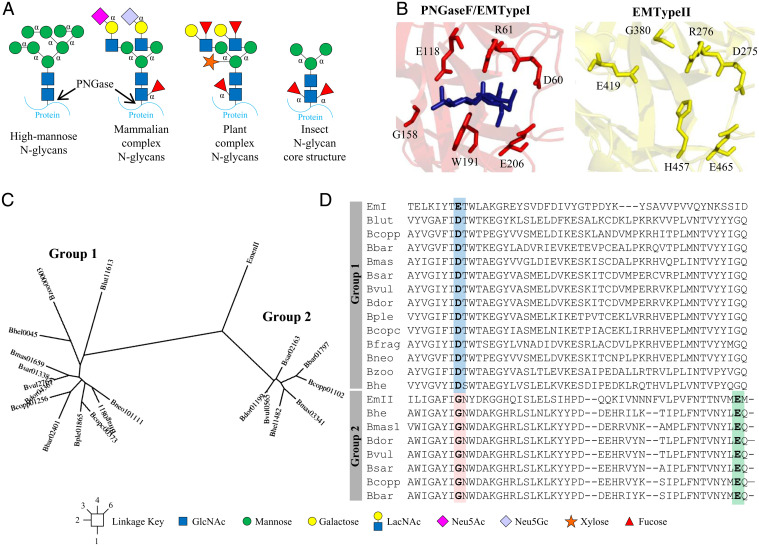
PNGases in species of *Bacteroides*. (*A*) Different types of *N*-glycans. High-mannose *N*-glycans (HMNGs) have mannose sugars decorating both arms usually to give a total of between five and nine mannose sugars, dubbed Man5 and Man9, respectively, for example. HMNGs do not vary between different organisms, whereas complex *N*-glycans do have differences according to the source. In mammals, complex *N*-glycans have LacNAc disaccharides (Galβ1,4GlcNAc) attached to the mannose arms through a β1,2-linkage. The galactose sugars are typically decorated with sialic acids, but these can also decorate the antenna GlcNAc. Mammalian complex *N*-glycans can have additional antenna through a β1,4-linkage on the α1,3-mannose arm and a β1,6-linkage on the α1,6-mannose arm to produce tri- and tetra-antennary structures, respectively. An α1,6-fucose is a common decoration on the first core GlcNAc in mammals, but α1,3/4-linked fucose is also found to decorate the antenna GlcNAc. In contrast, plant *N*-glycans typically have Lewis A epitopes (Galβ1,3[Fucα1,4]GlcNAc) as their antenna, a core α1,3-fucose, and a bisecting β1,2-xylose. Insect *N*-glycan structures typically have both α1,3- and α1,6-fucose decorating the core GlcNAc. (*B*) Active sites of the two PNGases from *E. meningoseptica*. The key active site residues are shown as sticks and chitobiose is present in the PNGaseF/EMTypeI structure. (*C*) Phylogenetic tree of the PNGase enzymes from *Bacteroides* species, which broadly split into two groups. The members of group 1 have quite variable identity between them, as low as 52% in one instance, but generally between 67 and 99%. The members of group 2 have 75 to 97% identity between them. The codes used for the different species is explained Fig. S2. (*D*) A sequence alignment to show residues that are key to the specificity of accommodating the core α1,3-fucose often present on plant *N*-glycans. The residue blocking the α1,3-fucose binding in the group I PNGases is highlighted in blue (E118 in PNGaseF/EMTypeI; Asp in the *Bacteroides* enzymes) and the glycine replacing this residue in the group 2 PNGases is highlighted in pink (G380 in EMTypeII). The glutamic acid replacing the function of E118 is highlighted in green (E419 in EMTypeII).

*N*-glycans are common decorations of secreted proteins from almost all types of organisms and play important roles in protein stability and function (3). Although the core structures of plant and mammalian *N*-glycans are conserved, key differences exist in the types of sugar decorations and linkages. As a broad classification, mammalian complex *N*-glycans commonly have an α1,6-fucose linked to the base *N*-acetylglucosamine (GlcNAc) whereas plants frequently have an α1,3-fucose on this sugar and a β1,2-xylose linked to the first mannose (Fig. 1). Insect *N*-glycans commonly have both α1,3- and α1,6-fucose linked to the core *N*-glycan. Plant complex *N*-glycans also differ from mammalian complex *N*-glycans in their antennae structures, which have β1,3-galactose and α1,4-fucose decorating the GlcNAcs (Fig. 1) (4), although there is significant variation in plant *N*-glycan structures depending on the species (5). Plant *N*-glycans are also of interest as they can be highly antigenic and induce allergic responses in mammals, causing both hayfever and food allergies (6–8). Furthermore, when plants are used as hosts for heterologous protein production for medical applications, the *N*-glycans present on these therapeutic proteins can pose a risk to function and stability. Thus there is a need to be able to both characterize and modify plant *N*-glycans from different sources for a range of applications. Plant *N*-glycan acting CAZymes would be useful tools for this job, but currently there is a paucity of data describing enzymes that act specifically on plant *N*-glycan structures.

Previous studies investigating carbohydrate-degradation systems in gut bacteria have typically used transcriptomics during growth on a specific glycan to identify the PUL or PULs involved in its breakdown (9, 10). These experiments require relatively large amounts of substrate for the bacteria to grow on, but for some glycans it may not be possible to isolate enough material, which means that discovery of enzymes that act on these substrates is currently limited. Plant *N*-glycans are a good example as although these molecules are common components of plant material and therefore widely consumed in the human diet, they are not easily available in the amounts required for transcriptomic studies (9). Here we describe a genome mining approach—“PULomics”—to look for the enzyme apparatus in *Bacteroides* species that degrade complex plant *N*-glycans. We relied on information about activity and specificity for particular enzyme families, assessed them for activity, and also extended this analysis to neighboring putative CAZyme genes. We describe the biochemical characterization of five CAZymes and two crystal structures to provide a degradation pathway for plant complex *N*-glycans encoded by the human gut microbiome. The study provides a toolbox of activities that will allow modification of plant and insect *N*-glycan structures for biotechnological and medical applications.

## Results

### Bioinformatics Analysis Shows Two Types of PNGase Are Present in *Bacteroides* Species.

There are currently two main classes of enzyme that remove *N*-glycans from glycoproteins: glycoside hydrolases (from either GH18 or GH85 families) and peptide-*N*^4^-(*N*-acetyl-β-glucosaminyl)asparagine amidases (PNGases). The GH18 and GH85 enzymes hydrolyze the β1,4 glycosidic bond between the two core GlcNAc sugars, whereas PNGase family members cleave the linkage between the first GlcNAc and Asn of the protein/peptide. PNGaseF (or EMTypeI) from *Elizabethkingia meningoseptica* is widely used to remove mammalian-type *N*-glycans for analysis, which commonly have an α1,6-fucose attached to the first core GlcNAc (known as “type I” activity) (11). However, this enzyme is not able to accommodate *N*-glycan structures with α1,3-fucose attached to the core GlcNAc that are common decorations in plant and insect *N*-glycans (11). More recently, a “type II” PNGase also from *E. meningoseptica* was characterized that displayed additional activity toward *N*-glycans with a core α1,3-fucose (EMTypeII) (11). Structures of both type I and II PNGase enzymes exist allowing insight into how these different α-fucose decorations are accommodated or blocked (11). In PNGaseF/EMTypeI, the active site residue Glu118 blocks where an α1,3-fucose may have potentially been accommodated, whereas in the EMTypeII structure, the equivalent residue is a glycine (G380), creating a pocket for this sugar (Fig. 1*B*). Glu418 in EMTypeII likely carries out the equivalent coordinating role to Glu118 in EMTypeI (11).

Recent work with *B. thetaiotaomicron* has revealed the pathways of degradation of mammalian-type complex and high-mannose *N*-glycans by this gut symbiont (9, 12). Degradation of both of these *N*-glycan types involves GH18 endo-β-GlcNAc-ase activity to remove the glycan from the protein. *B. fragilis* has also been shown to degrade complex mammalian-type *N*-glycans (13), and a number of other *Bacteroides* species have the capacity to grow on glycoproteins with complex *N*-glycan structures (9). We wanted to further investigate the *N*-glycan degradation capacity of gut *Bacteroides* species further by exploring the prevalence and function of putative PNGases encoded by these prominent symbionts.

Analysis of the Integrated Microbial Genomes and Microbiomes system (IMG/M) (14) revealed the presence of putative PNGases in 13 species of *Bacteroides*, with 7 of these species having two PNGase genes each. These PNGase sequences clustered broadly into two groups according to sequence identity (Fig. 1*C* and *SI Appendix*, Table S1). Sequence alignment included comparison to PNGases from *E. meningoseptica* and revealed an interesting trend in terms of the possible substrate preferences of the two groups of PNGases (Fig. 1*D*). Group 1 enzymes have conserved aspartate residues in the equivalent position to Glu118 in EMTypeI, whereas Group 2 enzymes all had glycine at this position (Fig. 1*D*), as has been highlighted previously in a comparison between PNGaseF/EMTypeI and EMTypeII (11). This indicated that group 1 and group 2 *Bacteroides* PNGases may have similar substrate preferences to PNGaseF/EMTypeI and EMTypeII, respectively, in terms of their ability to accommodate a core α1,3-fucose. Structures for the group 1 PNGases from *B. fragilis* and *B. vulgatus* are also available and confirm a similar positioning of the key catalytic residues relative to PNGaseF/EMTypeI (*SI Appendix*, Fig. S1).

Another striking difference between the two groups is the presence of an N-terminal domain in most of the group 2 protein sequences (all except *B. coprophilus*) and not in the group 1 sequences (*SI Appendix*, Fig. S2). This N-terminal domain is present in EMTypeII, but not PNGaseF/EMTypeI, and has previously been dubbed the N-terminal bowl-like domain (NBL) (11). The NBL domain has a unique structure and unknown function, but the expression of the catalytic domain alone without the NBL did not affect activity or specificity of EMTypeII (11).

A final notable difference between the two PNGase groups is a small insert between the two eight-stranded antiparallel β-sheets in the sequences in group 1 (*SI Appendix*, Fig. S2). In the two available crystal structures of group 1 *Bacteroides* PNGases from *B. fragilis* and *B. vulgatus*, this translates into a small β-sheet twist adjacent to the active site (*SI Appendix*, Fig. S1), although interestingly PNGaseF/EMTypeI does not have this insert. Further analysis of the crystal structures of the PNGases from *B. fragilis* and *B. vulgatus* reveals that in both cases these enzymes crystallized as dimers in the asymmetric unit with the β-sheet twist being the major interaction point. Notably the active sites were not blocked by this dimerization (*SI Appendix*, Fig. S1), suggesting the dimer could be functionally relevant.

### The Activity of Group 1 and 2 PNGases from *Bacteroides* species.

**Fig. 2.**
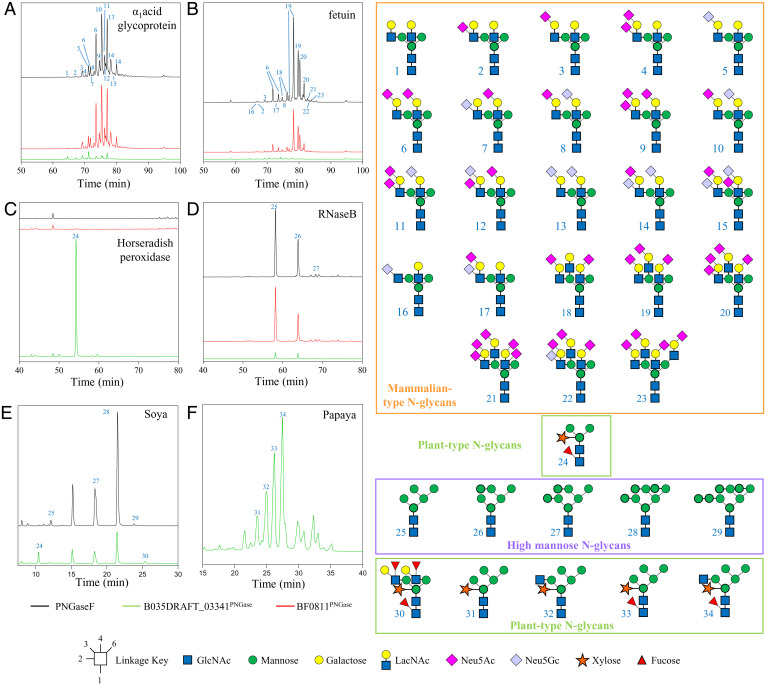
Activity of PNGase B035DRAFT_03341 from *B. massiliensis* and PNGase BF0811 from *B. fragilis* against different substrates. (*A*) α_1_acid glycoprotein. (*B*) Fetuin. (*C*) Horseradish peroxidase. (*D*) RNaseB. (*E*) Soya protein. (*F*) Papaya protein. B035DRAFT_03341 (green), PNGaseF (black), and BF0811 (red). The time window shown for the different chromatograms varies between the panels to provide clarity of the main peaks. The glycan products for *A*–*E* were labeled with procainamide and analyzed by LC-FLD-ESI-MS. The glycan products for *F* were labeled with 2-aminobenzamide (2-AB) and analyzed by UPLC.

To explore the activity of the PNGases from *Bacteroides* species, a PNGase from each group (BF0811^PNGase^, a group 1 enzyme from *B. fragilis*, and B035DRAFT_03341^PNGase^, a group 2 enzyme from *B. massiliensis*) was tested for activity against glycoprotein substrates displaying a range of different *N*-glycan structures. These included fetuin, α_1_acid glycoprotein, RNaseB, and horseradish peroxidase (HRP), which have predominantly triantennary complex, biantennary complex, high mannose, and plant-type *N*-glycan decorations, respectively (Fig. 2). Commercially available PNGaseF/EMTypeI was also assayed for comparison. Released *N*-glycans were subsequently labeled with procainamide and analyzed by liquid chromatography–fluorescence detection–electrospray ionization–mass spectrometry (LC-FLD-ESI-MS). The *B. fragilis* PNGase from group 1 (BF0811^PNGase^) displayed very similar activity to PNGaseF/EMTypeI with the removal of complex and high-mannose *N*-glycans, but not plant *N*-glycans (Fig. 2). In contrast, the *B. massiliensis* PNGase from group 2 (B035DRAFT_03341^PNGase^) showed good activity toward plant-type *N*-glycans on HRP (Fig. 2C), with negligible activity against other structures (Fig. 2).

To further explore the substrate preference of B035DRAFT_03341^PNGase^, we used soya and papaya protein extracts as substrates (Fig. 2 and *SI Appendix*, Figs. S3 and S4). The results demonstrated the ability of B035DRAFT_03341^PNGase^ to remove more decorated plant *N*-glycans, including those with high-mannose, hybrid, and complex antennary structures. For the soya protein, PNGaseF/EMTypeI was unable to remove structures with a core α1,3-fucose (Fig. 2*E*, peak 24 is missing).

We also tested the activity of B035DRAFT_03341^PNGase^ against honey bee venom glycoprotein phospholipase A_2_, as activity against insect *N*-glycans (have both α1,3 and α1,6 core fucose) has previously been observed for EMTypeII PNGase (11). A decrease in molecular weight of phospholipase A_2_ was observed with the addition of B035DRAFT_03341^PNGase^ and there was also a decrease in intensity when staining for glycoproteins, indicating removal of *N*-glycan from this substrate by the enzyme (*SI Appendix*, Fig. S5).

Overall these data show that B035DRAFT_03341^PNGase^ requires a core α1,3-fucose for optimal activity (i.e., plant *N*-glycans), but that the presence of an α1,6-fucose can be accommodated (activity against insect *N*-glycans). This is slightly different to the specificity observed for EMTypeII PNGase, which does have activity against mammalian *N*-glycans, although the two PNGases were not directly compared in this work.

Notably, the two *Bacteroides* PNGases are predicted to have a type I signal sequence, indicative of localization to the periplasm (*SI Appendix*, Table S2), which would suggest that deglycosylation occurs after the substrate has been imported across the outer membrane. This would be in contrast to GH18-directed cleavage of *N*-glycans which occurs at the cell surface in *B. thetaiotaomicron* (9, 12). One potential reason for this may be that the preferred PNGase substrates are smaller glycopeptides that are products of proteolytic digestion of glycoproteins and so can be easily transported across the outer membrane, whereas GH18 enzymes act against the larger native glycoprotein, thus precluding import prior to deglycosylation. However, it is also possible that the signal sequences prediction is incorrect and the PNGases are localized to the outside of the cell, as has been seen previously (9).

### The Structure of the PNGase from *B. massiliensis*.

**Fig. 3.**
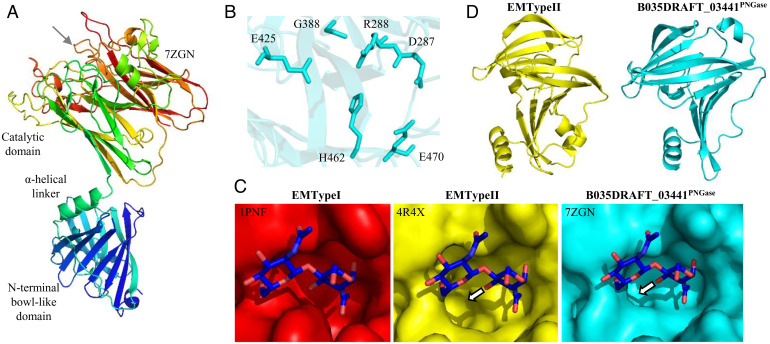
Structure of the group 2 PNGase from *B. massiliensis*. (*A*) Structure of the group 2 PNGase from *B. massiliensis* with two different domains. The protein is shown in a rainbow gradient with the N and C termini going from blue to red, respectively. The position of the active site is indicated (gray arrow). (*B*) Key catalytic residues of the active site. (*C*) Surface representation of the active sites of three PNGases to show the space to accommodate α-1,3-fucose in EMTypeII and B035DRAFT_03441^PNGase^. The arrows indicate the C3 of the GlcNAc where the α1,3-fucose would be attached, and the direction indicates the space it would approximately occupy. For EMTypeI there is no equivalent space where the α1,3-fucose would sit. EMTypeI had chitobiose crystallized in the active site and this is overlaid. (*D*) Two NBL domains from EMTypeII and B035DRAFT_03441^PNGase^ to show their structural similarity.

To investigate the structural basis for specificity in the *Bacteroides* PNGase enzymes from group 2, we solved the crystal structure for B035DRAFT_03341^PNGase^ to 1.95 Å (*SI Appendix*, Table S3). The structure consists of two domains: the catalytic domain and an NBL domain, which are linked through a flexible α-helical linker (Fig. 3*A*). The catalytic domain consists of two eight-stranded anti-parallel β-sheets, which is a consistent structural feature of PNGase enzymes described so far. The active site residues that are key for activity in EMTypeII are conserved in B035DRAFT_03341^PNGase^, with G388 occupying a critical position to allow the accommodation of α1,3-fucose (Fig. 3*B*). The available space for this fucose is particularly apparent in comparison to PNGaseF/EMTypeI (Fig. 3*C*). In contrast, the α1,6-fucose points away from the active site, so is not an issue in terms of blocking activity, consistent with the biochemical data. The NBL domain in the *B. massiliensis* structure has a very similar fold to the one in EMTypeII and is unique to these two proteins. They consist of 11 β-sheets connected by short α-helical regions and disordered loops (Fig. 3*D*). The difference in activities between B035DRAFT_03341^PNGase^ and EMTypeII has been explored in the context of their structures in the *SI Appendix*, *Results and Discussion* and *SI Appendix*, Fig. S6.

### *B. massiliensis* Also Encodes an α1,3-Mannosidase Able to Accommodate the Plant *N*-Glycan β1,2-Xylose Decoration.

**Fig. 4.**
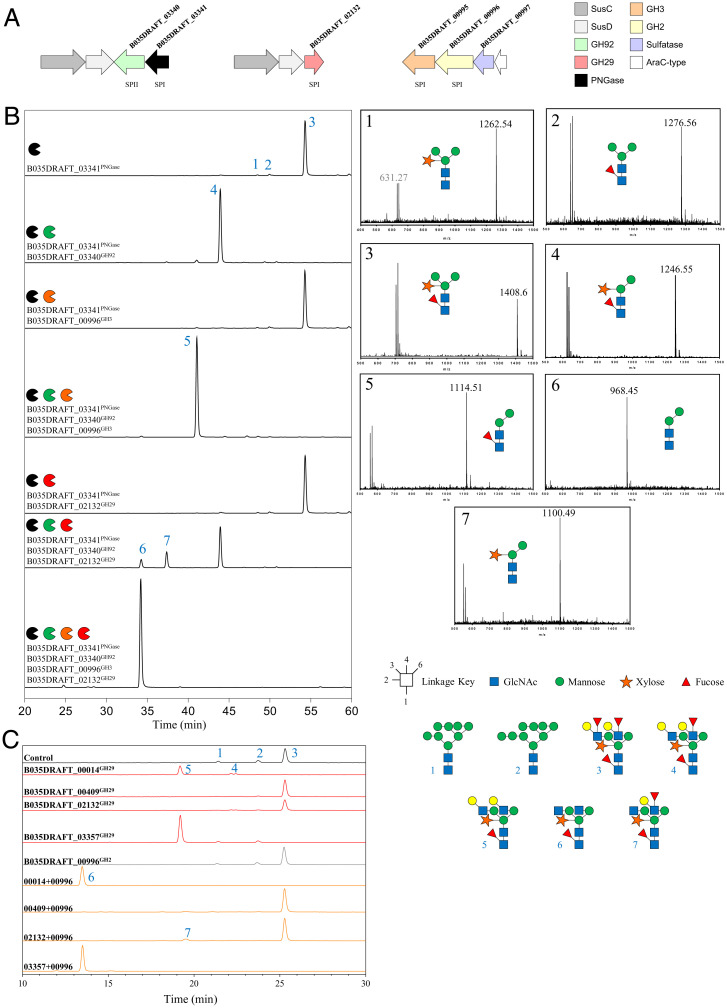
Activity of CAZymes identified from the functional association analysis against plant N-glycan structures. (*A*) Genes identified encoding the putative CAZymes highlighted by the functional association analysis carried out in search of enzymes involved in the degradation of plant *N*-glycans. Predicted signal peptide types are shown below the CAZymes. (*B*) Horseradish peroxidase was incubated with different combinations of enzymes, and the products were labeled with procainamide at the reducing end and analyzed by LC-FLD-ESI-MS (*Left*). The mass spectra of the different peaks are provided (*Right*). (*C*) Activity of *B. massiliensis* GH29 putative α-fucosidases against soya bean *N*-glycans in the presence (orange) and absence (red) of B035DRAFT_00996^GH2^ galactosidase (gray indicates galactosidase only).

The presence of type II plant *N*-glycan active PNGase enzymes in prominent members of the human gut microbiota suggests that these microbes use plant *N*-glycans as a nutrient source. To identify the other enzymes required to fully degrade these glycans, we first examined the putative CAZyme genes adjacent to the PNGase genes in a number of different gut *Bacteroides* species. The B035DRAFT_03341^PNGase^ gene is next to a putative GH92 (B035DRAFT_03340^GH92^) in *B. massiliensis*, the characterized members of which are all α-mannosidases (Fig. 4*A*).

Incubation of this recombinant enzyme with α-mannobiose of varying linkages showed activity against the α1,3-linked disaccharide only (*SI Appendix*, Fig. S7). B035DRAFT_03340^GH92^ was then assayed against HRP, which has complex plant *N*-glycans, although these lack the extensive antennary decorations seen in other plant *N*-glycans. B035DRAFT_03340^GH92^ was able to remove the α1,3-mannose from HRP *N*-glycan heptasaccharide once it was removed from the protein and also while the glycan was still attached to the protein (Fig. 4*B* and *SI Appendix*, Fig. S7).

To further study the specificity of B035DRAFT_03340^GH92^ it was compared to a GH92 that has specificity toward the α1,3-mannose linkages in high mannose *N*-glycans (BT3991^GH92^ from *B. thetaiotaomicron*) (12). BT3991^GH92^ was unable to remove mannose from HRP either with or without the removal of the *N*-glycan from the protein, whereas B035DRAFT_03340^GH92^ could cleave the α1,3-mannose from both free HRP glycan or while the *N*-glycan was attached to protein (*SI Appendix*, Fig. S7). The lack of activity seen for BT3991^GH92^ is likely due to the plant-specific β1,2-xylose on the core mannose causing steric hindrance within the active site, whereas B035DRAFT_03340^GH92^ is able to accommodate this decoration.

Close homologs of B035DRAFT_03340^GH92^ are found in all of the *Bacteroides* species analyzed that have a group 2 PNGase encoded in the genome (*SI Appendix*, Fig. S8). These homologs had identity between 77 and 96% (*SI Appendix*, Table S4) and are present adjacent to the gene for the group 2 PNGase for *B. massiliensis*, *B. vulgatus*, and *B. helcogenes*, but not in the case of *B. dorei*, *B. sartorii*, *B. coprophilus*, or *B. barnesiae* (*SI Appendix*, Fig. S8). An exception is *B. neonati* that also has a homolog of B035DRAFT_03340^GH92^ with 73% identity, but no putative group 2 PNGase gene and, unexpectedly, this GH92 gene is adjacent to the putative group 1 PNGase in *B. neonati* (*SI Appendix*, Fig. S8). B035DRAFT_03340^GH92^ has a type II signal sequence indicating it is membrane-associated, although whether the enzyme is localized to the cell surface or faces the periplasm was not experimentally determined.

### Crystal Structure of the GH92 α1,3-Mannosidase.

**Fig. 5.**
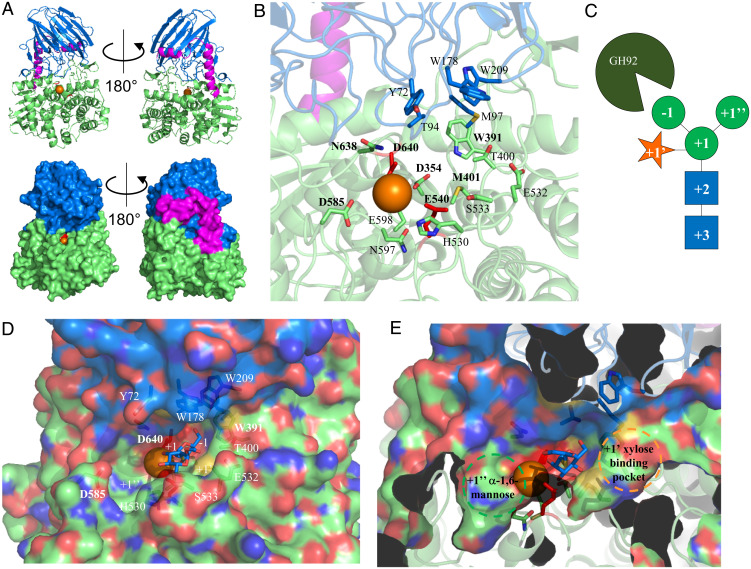
Crystal structure of the α1,3-mannosidase B035DRAFT_03340^GH92^. (*A*) Structures of B035DRAFT_03340^GH92^ shown in cartoon and surface (*Top* and *Bottom*, respectively). The N-terminal β-sandwich domain, the two connecting helices, and the C-terminal (α/α)_6_-barrel are shown in marine, magenta, and lime, respectively. The metal ion is shown in orange, and catalytic residues are shown in red. (*B*) Details of the active site with likely important residues shown as sticks. Those labeled in bold are conserved throughout the GH92 family, and those not in bold are unique to this enzyme. (*C*) Diagram showing the sugar subsites for this enzyme. (*D*) Surface representation of the active site of the GH92 with partial transparency so the residues in sticks can also be viewed. Thiomannobioside from BT3990 (PDB 2WW1) has been overlaid (blue sticks) to show the approximate −1 and +1 subsites. The likely +1′ xylose and +1″ mannose subsites are also labeled. (*E*) This is the same as *D*, but showing a cross-section through the front of the enzyme to show the extent of the binding pockets for the +1′ xylose and +1″ mannose subsites.

To investigate the structural basis for the unusual specificity displayed by B035DRAFT_03340^GH92^ we determined the crystal structure of the enzyme to 1.43 Å (*SI Appendix*, Table S3). The GH92 consists of two domains: an N-terminal β-sandwich domain composed of 16 antiparallel β-strands domain and an (α/α)_6_-barrel catalytic domain. These two domains are pinned together by two α-helices, previously dubbed helix 1 and 2 (Fig. 5*A*) (15). The secondary structures of the seven GH92 enzymes with known structures also have these three features: the N-terminal domain, the catalytic domain, and the α-helical linker. The density for several metal ions was observed bound to the protein surface, which were modeled as Na from the crystallization conditions, except for a Ca near the active site, as this metal has been shown to be key for GH92 activity.

The active site of B035DRAFT_03340^GH92^ is comprised of residues originating from both the N- and C-terminal domains, which is a common feature of the other GH92 structures (Fig. 5*B*). The residues that are conserved within the catalytic site throughout all GH92 enzymes with crystal structures derive from the C-terminal domain. Those residues that vary come from both the N- and C-terminal domains and are the drivers of specificity by interacting with the +1 subsite sugar and beyond (Fig. 5*C*). To explore the active site of B035DRAFT_03340^GH92^, we overlaid the nonhydrolyzable substrate mimic thiomannobioside from the structure of BT3990 (Protein Data Bank [PDB] 2WW1) (Fig. 5*D*). From this we could speculate about the location of four subsites, including the −1 α1,3-mannose, +1 core mannose, +1′ xylose and the +1″ α1,6-mannose (Fig. 5*C*). The environment of the −1 mannose is identical to that described for other GH92 enzymes structures (15–17). The +1 mannose is likely recognized by the side chains of at least three residues: H530, S533, and E532, which are positioned underneath the mannose. Overhanging this subsite (and possibly the +2 GlcNAc position also) are three hydrophobic residues Y72, W178, and W209, which is suggestive of π-stacking of the sugars. However, these aromatics are more distant than equivalent residues seen in other GH92 structures (15). The +1 subsite interactions allows space either side of the +1 mannose for the +1″ mannose and +1′ xylose. There are clear pockets for these sugars in the B035DRAFT_03340^GH92^ structure (Fig. 5*E*).

The eight GH92 structures already available include five α1,2-mannosidases, an α1,3-mannosidase, an α1,4-mannosidase, and a mannose-α1,4-PO_4_-mannose mannosidase (15–19). Previous comparison of the α1,2-mannosidase structures revealed three residues coordinating the mannose at the +1 subsite that drive specificity for α1,2-linkages. These are a Trp from the N-terminal domain and a Glu and His from the C-terminal domain, and these are also predicted through sequence alignments to be present in other GH92 α1,2-mannosidases. SP2145 from *Streptococcus pneumoniae* (PDB 5SW1) was crystallized with a mannose in the +1 subsite and demonstrates these interactions (*SI Appendix*, Fig. S9). In an attempt to highlight if there were any similar conserved motifs present for GH92 α1,3-mannosidases, we compared the structures of B035DRAFT_03340^GH92^ with BT3130 (PDB 6F8Z; *SI Appendix*, Fig. S9). This comparison saw no conservation in the active site residues associated with the +1 subsite. However, the residues contributed from the N-terminal domain were tryptophans, similar to those seen in the α1,2-mannosidases, but the location and orientation differed (*SI Appendix*, Fig. S9). Notably, in B035DRAFT_03340^GH92^ this tryptophan +1 subsite “lid” is much farther away from where the glycan would sit than in other GH92 structures. This lid would possibly reach down farther if substrate was present.

We carried out phylogenetic analysis of the GH92 enzymes that had been characterized to see if they would cluster according to their activities (*S**I Appendix*, Fig. S10). This was successful in that α1,2-mannosidases and α1,3-mannosidases clustered together, although it should be noted that the sequences were predominantly derived from *B. thetaiotaomicron*.

### Gene Association Analysis to Identify Additional Plant *N*-Glycan Degrading Enzymes from *Bacteroides* Species.

The characterization of PNGase homologs from gut *Bacteroides* species revealed there are likely two different PNGase-like activities encoded by these microbes: group 1 targeting mammalian *N*-glycans and group 2 targeting plant/insect *N*-glycan structures. We were also able to identify an α1,3-mannosidase with specificity toward complex plant *N*-glycans by characterizing the product of a GH92 gene associated with the group 2 PNGase gene in *B. massiliensis*. Genes in the same locus likely have functional associations and this is common in carbohydrate degradative systems in Bacteroidetes. We therefore expanded this concept to identify other putative plant *N*-glycan targeting CAZymes from *Bacteroides* species.

The group 2 PNGases from *B. dorei*, *B. barnesiae*, and *B. coprophilus* are all orphan genes (i.e., no obvious adjacent genes) and the PNGase from *B. sartorii* is only neighbors with a *sus*C/D pair; however, the group 2 PNGase genes from *B. helcogenes* and *B. vulgatus* all look to be a part of more extensive loci (*SI Appendix*, Fig. S8). The neighboring open reading frames (ORFs) included putative SusC/D pairs, GH29, GH3, GH130, and additional GH92 enzymes (*SI Appendix*, Fig. S8). Using this initial survey, a network of possible functionally related ORFs was built for all of the *Bacteroides* species with group 2 PNGase enzymes (*SI Appendix*, Fig. S8). Using this approach, we were able to highlight CAZymes with potential specificity toward plant *N*-glycans. For *B. massiliensis*, these CAZymes were located in two further putative loci (Fig. 4*A*). One locus consists of a *sus*C/D pair and a putative GH29 and the second has a GH3, a GH2, a sulfatase, and an AraC-type transcriptional regulator (Fig. 4*A*). The activities of the CAZymes from these *B. massiliensis* loci were then explored.

### B035DRAFT_00995^GH3^ Is a β-Xylosidase Acting on Plant *N*-Glycans.

A unique feature of plant *N*-glycans is the β1,2-xylose decoration on the core mannose (Fig. 1*A*). Some GH3 family enzymes are known to display β-xylosidase activity, but are mainly against β1,4-linkages in plant xylans (20). To investigate the specificity of the GH3 from *B. massiliensis*, B035DRAFT_00995^GH3^, a recombinant form of the enzyme, was screened against a variety of *p*-nitrophenyl (pNP) substrates and found to be active against pNP–β-xylose only. This enzyme was then tested with the plant-type heptasaccharide released by B035DRAFT_03341^PNGase^ (Fig. 4*B*). This was unsuccessful at removing the bisecting β1,2-xylose. However, when the reaction was carried out also in the presence of B035DRAFT_03340^GH92^ to remove the α1,3-mannose, B035DRAFT_00995^GH3^ was able to cleave this xylose (Fig. 4*B* and *SI Appendix*, Fig. S11). B035DRAFT_00995^GH3^ has a type I signal peptide and so is likely localized to the periplasm (*SI Appendix*, Table S2).

B035DRAFT_00995^GH3^ activity was also assessed against β1,4-xylobiose (*SI Appendix*, Fig. S11). No activity was observed, which indicates specificity for this enzyme toward β1,2-xylose linkages in plant *N*-glycans. For comparison, a previously characterized GH3 β-xylosidase from *B. ovatus* (BACOVA_03419) that is involved in the degradation of plant cell wall xylans (20) could hydrolyze β1,4-xylobiose (*SI Appendix*, Fig. S11).

Putative GH3 β1,2-xylosidases were identified for five out of the seven *Bacteroides* species with group 2 PNGase enzymes. Homologs of B035DRAFT_00995^GH3^ in *B. coprophilus*, *B. barnesiae*, *B. vulgatus*, and *B. helcogenes* have 66, 67, 75, and 76% identity, respectively (*SI Appendix*, Table S5). No obviously equivalent B035DRAFT_00995^GH3^ homologs could be identified in *B. dorei* or *B. sartorii* using gene association analysis.

### B035DRAFT_02132^GH29^ Is an α1,3-Fucosidase Active Against Core Decorations of Plant *N*-Glycans.

The fucose decorating the core GlcNAc of plant *N*-glycans is through an α1,3-linkage, in contrast to the α1,6-linkage of mammalian-derived *N*-glycans (Fig. 1*A*). Another enzyme identified through the functional association analysis was B035DRAFT_02132^GH29^ (*SI Appendix*, Fig. S12), which is predicted to be localized to the periplasm (*SI Appendix*, Table S2). GH29 family members typically have exo α1,3/4-fucosidase activities, so B035DRAFT_02132^GH29^ was screened against a variety of fucose-containing glycans (*SI Appendix*, Fig. S12). B035DRAFT_02132^GH29^ was found to only hydrolyze the α1,3-fucose from Lewis X trisaccharide to completion overnight, which is the glycan most similar to the core of a plant *N*-glycan out of the defined oligosaccharides that were tested. As a comparison it was only partially active against the α1,3-fucose from 3-fucosyllactose, which confirms a specificity for GlcNAc over Glc in the +1 subsite. Furthermore, B035DRAFT_02132^GH29^ was not able to remove the α1,4-fucose from Lewis A, which indicates that it does not target the antennary structures of plant *N*-glycans (*SI Appendix*, Fig. S12).

**Fig. 6.**
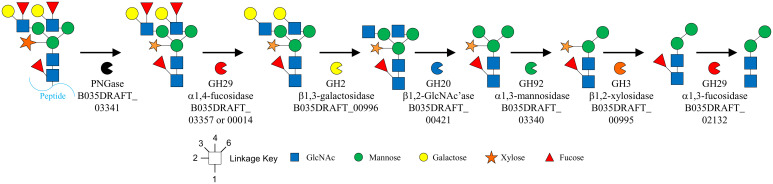
Likely degradation pathway for plant *N*-glycans by *B. massiliensis*. Schematic of the order in which *B. massiliensis* likely degrades plant *N*-glycans based on the biochemical data presented in this study.

When B035DRAFT_02132^GH29^ was tested against plant *N*-glycan heptasaccharide released by B035DRAFT_03341^PNGase^, no core fucose removal was observed (Fig. 4*B*). However, partial removal of the core α1,3-fucose was seen once the α1,3-mannose had been removed by B035DRAFT_03340^GH92^, and full removal of the α1,3-fucose by the GH29 was made possible after removal of the β1,2-xylose by B035DRAFT_00995^GH3^ (Fig. 4*B*). These observations provide insights into the likely plant *N*-glycan degradation pathway in *B. massiliensis* (Fig. 6). Homologs of these enzymes were in five out of the seven *Bacteroides* species with type II PNGase enzymes (*SI Appendix*, Table S6). There was no obvious homolog in *B. vulgatus* and *B. dorei* and there were also no obvious homologs in species without type II PNGases.

Fucosidases from different sources have previously been shown to act on the α1,3 core linkage of plant *N*-glycans. Most notably, a GH29 from *E. meningoseptica*, cFase I, can act on the core α1,3-fucose even when the plant *N*-glycan has antennary decoration (21). Another GH29 from *Arabidopsis thaliana*, AtFUC1, was also able to act on the α1,3-linkage, but only when the glycan was reduced down to an α1,3-fucose linked to chitobiose. A knockout of the AtFUC1 gene led to an accumulation of this trisaccharide in the plant confirming the specificity of enzymes toward the core fucose of plant *N*-glycans (22).

### B035DRAFT_00996^GH2^ Is a β1,3-Galactosidase that Targets the Antenna Decorations of Complex Plant *N*-Glycans.

The final CAZyme identified in *B. massiliensis* using the functional association analysis was a GH2, B035DRAFT_00996^GH2^ (*SI Appendix*, Fig. S12), which was screened against a variety of pNP substrates and found to be active against pNP–β-galactose. The enzyme was then screened against defined oligosaccharides to determine its substrate specificity in more detail (*SI Appendix*, Fig. S13). B035DRAFT_00996^GH2^ only had activity toward β1,3-linked galactose when GlcNAc was in the +1 position (lacto-*N*-biose). It could also act on lacto-*N*-tetraose, which has the same linkage and +1 sugar. It was unable to hydrolyze *N*-acetyllactosamine (LacNAc) or Galβ1,3Glc, which demonstrates the specificity toward the β1,3-linkage and a requirement for the *N*-acetyl group of the +1 GlcNAc, respectively. Furthermore, partial activity was observed toward Galβ1,3GalNAcβ1,3Galβ1,4Glc, which emphasizes the importance of the *N*-acetyl group in the +1 sugar with some influence also coming from the C4 hydroxyl of the +1 sugar either being axial or equatorial (Gal or Glc, respectively). These results show that B035DRAFT_00996^GH2^ β-galactosidase has specificity for the β1,3 linkage and +1 GlcNAc sugar found on the antenna of complex plant *N*-glycans.

Activity was also tested against Lewis A trisaccharide, which is the epitope of the antenna structure present on plant *N*-glycans (Fig. 1*A* and *SI Appendix*, Fig. S13). B035DRAFT_00996^GH2^ was unable to remove the galactose in this case, which suggests that a fucosidase must act before this galactosidase in the breakdown of the full plant *N*-glycan substrate. Analysis of the galactosidase activity against a soya-derived complex plant *N*-glycan structure in the presence of α-fucosidases (see below) confirmed that the Gal decorations could only be removed after the antennary fucoses have first been cleaved (Fig. 4*C*). B035DRAFT_00996^GH2^ is predicted to be periplasmic (*SI Appendix*, Table S2).

Notably, this galactosidase did not have close homologs in other *Bacteroides* species and was highlighted in *B. massiliensis* by its association with B035DRAFT_00995^GH3^ xylosidase. Genes encoding putative β1,3-galactosidases in other *Bacteroides* species with group 2 PNGase enzymes were not obvious from the functional association analysis, suggesting the terminal galactose structures are likely targeted by a CAZyme unrelated to this GH2 in these other species.

### Antennary Fucose Removal from Plant *N*-Glycans.

Complex plant *N*-glycans are also often decorated with antennary α1,4-fucose (Fig. 1*A*). GH29 enzymes typically act on α1,3/4-linkages, but there are some examples of α1,2-specific enzymes. Only a single GH29 was identified in *B. massiliensis* using functional association analysis (B035DRAFT_02132^GH29^), and this was shown to be specific for the core α1,3 fucose. Therefore, to test the possibility of this species being able to degrade the antennary fucose structures we screened the activity of three further GH29 enzymes from *B. massiliensis*. All three displayed relatively broad activity against Lewis and fucosyllactose glycans (*SI Appendix*, Fig. S13). In particular, all three were able to hydrolyze the α1,4-fucose from Lewis A trisaccharide, which is the epitope found in plant complex *N*-glycans, whereas B035DRAFT_02132^GH29^ was unable to do this.

The GH29 fucosidases were then assessed against soya bean–derived *N*-glycans (Fig. 4*C*). B035DRAFT_00014^GH29^ and B035DRAFT_03357^GH29^ were able to remove the antennary fucose from the complex *N*-glycan structures. Interestingly, B035DRAFT_00409^GH29^ was unable to do this despite being active against Lewis A trisaccharide. Furthermore, incubation of B035DRAFT_00996^GH2^ β1,3-galactosidase against the soya bean *N*-glycans in combination with either B035DRAFT_00014^GH29^ or B035DRAFT_03357^GH29^ showed removal of the terminal galactose and confirms that either GH29 provides access to the galactose for B035DRAFT_00996^GH2^ on plant *N*-glycan structures.

Although this screen of GH29 activities in *B. massiliensis* is not exhaustive, it does demonstrate that there are multiple enzymes present in the genome with the capability to remove the antennary fucose from plant *N*-glycans.

Once the antennary fucose and galactose has been removed from the nonreducing end of a complex plant *N*-glycan structure, the β1,2-GlcNAcs will be the last antennary sugars left to remove (Fig. 1*A*). In a previous report, we described a set of GH20 enzymes from *B. thetaiotaomicron* with specificity toward these particular structures in mammalian complex *N*-glycans (9). Homologs of these GH20 enzymes in *B. massiliensis* were recombinantly expressed and tested against soya protein plant *N*-glycans after fucosidase and galactosidase treatment. One of these enzymes, B035DRAFT_00421^GH20^, was able to remove these antennary GlcNAc decorations (*SI Appendix*, Fig. S14 and *SI Appendix*, *SI Results and Discussion*).

## Discussion

In this study we characterize the likely pathway for the degradation of plant *N*-glycans by a prominent member of the human gut microbiota. The enzymes involved were identified through functional association using putative PNGases as a starting point. The work demonstrates that it is possible in some cases to find enzymes with particular activities without using gene up-regulation methods and only using what is already known about the specificity of CAZyme families. This is a useful demonstration because for many substrates, such as for the plant complex *N*-glycans described here, it is not possible to perform gene up-regulation studies to identify the link between a glycan and the set of genes that encode the enzymes that target its breakdown.

The specificity of the enzymes characterized here likely indicates the order in which they act in vivo on the plant *N*-glycan structures (Fig. 6). B035DRAFT_03341^PNGase^ removes the *N*-glycan from the protein, or more likely peptide, as the PNGase preferentially acts on the glycopeptide, and then monosaccharides are removed sequentially from the nonreducing end of the glycan, although it is possible some of the glycosidases can act on the glycan before removal by the PNGase. On complex plant *N*-glycans, any α1,4-linked antennary fucoses are first removed by GH29 fucosidases such as B035DRAFT_00014^GH29^ and B035DRAFT_03357^GH29^, which then allows B035DRAFT_00996^GH2^ to remove the terminal plant-specific β1,3-galactoses. This is followed by removal of antennary β-linked GlcNAc structures by GH20 enzymes such as B035DRAFT_00421^GH20^. B035DRAFT_03340^GH92^ is then able to remove the α1,3-mannose, followed by B035DRAFT_00995^GH3^ to cleave the plant-specific β1,2-xylose, and B035DRAFT_02132^GH29^ to release the core α1,3-fucose, leaving a Manα1,6Manβ1,4GlcNacβ1,4GlcNac tetrasaccharide. This structure is common to all *N*-glycans and thus likely targeted by alternative loci/pathways in *Bacteroides* species. There is currently no reported enzyme that can remove the α1,6-Man, but there are enzymes from the GH2 and GH130 families that are capable of removing the β1,4-Man (23, 24). We did not explore which GH20 family members from *B. massiliensis* would be able to hydrolyze the remaining chitobiose; however, we have previously reported this activity in the context of mammalian complex *N*-glycan breakdown by a GH20 enzyme from *B. thetaiotaomicron* (BT0459; 73% identity to B035DRAFT_00421^GH20^) (9). This activity is also not just restricted to the GH20 family.

In addition to providing understanding of how plant *N*-glycans are degraded in the human gut, we also showed that insect glycoproteins may also be used as a nutrient source for the human gut microbiota as B035DRAFT_03341^PNGase^ is also active against insect *N*-glycan structures. Insects have been a human food source for centuries for some populations, they are common in other primate diets, and there is an increased interest in the use of insect protein in human diets for environmental and sustainability reasons (25).

This report also provides tools for modifying proteins decorated with plant and insect *N*-glycans, such as biopharmaceuticals. One of the potential uses of the specific plant *N*-glycan targeting CAZymes identified in this study would be in the production of pharmaceutical proteins in different plant species. Successful examples of this type of production include antibodies (“plantibodies”), collagen, vaccines, and enzymes, which can be produced in maize, rice, tobacco, flax, or strawberry (26). Monoclonal antibodies are potent treatments for a number of human diseases, including cancer and COVID-19 (27). Variation in the composition of the *N*-glycans decorating the antibodies have been seen to affect the function of these biological therapeutics (28). Therefore, expanding the options for *N*-glycan modification postproduction has the potential to increase the success rate of different candidate therapeutics produced in plant and insect cells, as well as provide opportunities to reduce the allergenicity of plant-produced proteins.

## Materials and Methods

### Sources of Glycans and Glycoproteins.

Glycoproteins bovine α_1_acid glycoprotein, bovine fetuin, bovine RNaseB, horseradish peroxidase, bee venom phospholipase A_2_, and pNP monosaccharides were obtained from Sigma. Defined oligosaccharides were purchased from Carbosynth. The isolation of papaya and soya *N*-glycans is described in detail in *SI Appendix*, Materials and Methods.

### Bacterial Strains.

The *Bacteroides* strains used were *B. fragilis* NCTC9342 and *B. massiliensis* DSM17679. *B. fragilis* was grown on tryptone–yeast extract–glucose medium with the addition of hematin (29), and *B. massiliensis* was grown on chopped meat broth (30, 31), and both were inoculated from glycerol stocks. Genomic DNA was prepared using a 5-mL culture.

### Cloning, Expression, and Purification of Recombinant Proteins.

DNA encoding the appropriate genes (excluding the signal sequences) was amplified from genomic DNA using appropriate primers and cloned into pET28b (Novagen). Recombinant plasmids were transformed into Tuner cells (Novagen) in Luria-Bertani (LB) broth containing 10 μg/mL kanamycin at 37 °C shaking at 180 rpm. One-liter cultures were grown to midexponential phase in 2-L baffled flasks, cooled to 16 °C, and isopropyl β-d-thiogalactopyranoside (IPTG) was added to a final concentration of 0.2 mM. These cells were then incubated for 16 h at 16 °C in an orbital shaker at 150 rpm. Recombinant His-tagged protein was purified from cell-free extracts using immobilized metal affinity chromatography (IMAC using Talon resin; Clontech) as described previously (32). The purity and size of the proteins were checked using sodium dodecyl sulfate–polyacrylamide gel electrophoresis (SDS-PAGE) and their concentrations determined using absorbance at 280 nm (NanoDrop 2000c; Thermo Scientific) and their molar extinction coefficients (33).

### Recombinant Enzyme Assays.

The activities of the recombinant enzymes were typically assessed in 20 mM 4-morpholinepropanesulfonic acid (MOPS) (pH 7) at 37 °C, with a final glycoprotein concentration of 20 mg/mL and a final enzyme concentration of 1 μM. The bee venom phospholipase A_2_ assay was carried out at 0.5 mg/mL, with 4 μg loaded on a SDS-PAGE gel. The SDS-PAGE gel used was a precast 8 to 16% gradient (Bio-Rad) and initially stained using a Pro-Q Emerald 300 glycoprotein staining kit to highlight glycoproteins and subsequently stained with Coomassie Brilliant Blue to visualize total protein. For overnight assays, defined oligosaccharides were incubated at a final concentration of 1 mM in the presence of 3 μM enzyme.

### Thin-Layer Chromatography.

For defined oligosaccharides, 3 μL of an assay containing 1 mM substrate was spotted onto silica plates. For assays against glycoproteins, this was increased to 9 μL. The plates were resolved in running buffer containing butanol/acetic acid/water (2:1:1) and stained using a diphenylamine-aniline-phosphoric acid stain (34).

### Procainamide Labeling.

Procainamide labeling was performed by reductive amination using a procainamide labeling kit containing sodium cyanoborohydride as a reductant (Ludger). Excess reagents were removed with S cartridges (Ludger). Cartridges were conditioned successively with 1 mL of deionized (DI) water, 5 mL of 30% acetic acid (vol/vol), and 1 M acetonitrile. Procainamide-labeled samples were then spotted on the cartridge and allowed to adsorb for 15 min. The excess dye was washed with acetonitrile. Labeled *N*-glycans were eluted with 1 mL of DI water.

### LC-FLD-ESI-MS Analysis of Procainamide-Labeled Glycans.

Procainamide-labeled glycans were analyzed by LC-FLR-ESI-MS. Here, 25 µL of each sample (prepared in 24:76 water/acetonitrile solution) was injected into a Waters ACQUITY UPLC (ultra-performance liquid chromatography) Glycan BEH Amide column (2.1 × 150 mm, 1.7-µm particle size, 130-Å pore size) at 40 °C on a Dionex Ultimate 3000 UHPLC (ultra-high-performance liquid chromatography) instrument with a fluorescence detector (fluorescence excitation wavelength [λ_ex_] = 310 nm, fluorescence emission wavelength [λ_em_] = 370 nm) attached to a Bruker amaZon speed ETD. Mobile phase A was a 50 mM ammonium formate solution (pH 4.4), and mobile phase B was neat acetonitrile. Analyte separation was accomplished by gradients running at a flow rate of 0.4 mL/min from 85 to 57% mobile phase B over 105 min for α1acid glycoprotein and fetuin N-glycans and from 85 to 62% over 95 min for horseradish peroxidase and RNaseB samples, respectively. The amaZon speed was operated in the positive sensitivity mode using the following settings: source temperature, 180 °C; gas flow, 41 min^−1^; capillary voltage, 4,500 V; ICC target, 200,000; maximum accumulation time, 50.00 ms; rolling average, 2; number of precursor ions selected, 3; scan mode, enhanced resolution; mass range scanned, 400 to 1,700.

### Crystallization.

B035DRAFT_03341^PNGase^ and B035DRAFT_03340^GH92^ were initially screened using commercial kits (Molecular Dimensions, Qiagen, and Hampton Research). The protein concentrations were 20 mg/mL. The drops, composed of 0.1 or 0.2 µL of protein solution plus 0.1 µL of reservoir solution, were set up in a mosquito crystallization robot (SPT Labtech). The sitting drop method was used and the plates were incubated at 20 °C. The crystallization condition for B035DRAFT_03340^PNGase^ was condition B12 in JCSG screen part I (Qiagen). B035DRAFT_03341^GH92^ was condition E12 in Index screen (Hampton Research). The samples were cryoprotected with the addition of 20% polyethylene glycol (PEG) 400 to the crystallization condition.

### Data Collection, Structure Solution, Model Building, Refinement, and Validation.

Diffraction data were collected at the synchrotron beamlines I03 and I04 of a Diamond Light Source (Didcot, UK) at a temperature of 100 K. The dataset was integrated with dials (35) or XDS (36) via xia (37) and scaled with Aimless (38). The space group was confirmed with Pointless. The phase problem was solved by molecular replacement with Phaser (39) using PDB files 2WVX and 4R4Z as search models for B035DRAFT_03340^PNGase^ and B035DRAFT_03341^GH92^, respectively. While the initial solution Rfactors were very poor (over 50%) for B035DRAFT_03340^PNGase^, the electron density map was interpretable. The automated model building program task CCP4build on CCP4cloud (40) delivered a model with Rfactors below 20%. The models were refined with refmac (41) and manual model building with COOT (42). The final model was validated with COOT (42) and MolProbity (43). Other programs used were from the CCP4 suite (40). Processing and refinement statistics are reported in *SI Appendix*, Table S3. Models and data were deposited to the PDB with the codes 7ZGN and 7ZGM for B035DRAFT_03341^PNGase^ and B035DRAFT_03340^GH92^, respectively. Figures were made with PyMOL (44).

### Bioinformatics.

Putative signal sequences were identified using SignalP 5.0 (45). Sequence identities were determined using Clustal Omega using full sequences (46). The Integrated Microbial Genomes (IMG) database was used to analyze synteny between different species (47). The CAZy database (http://www.cazy.org) was used as the main reference for CAZymes (48). Alignments and phylogenetic trees were completed in SeaView (49). To determine the boundaries between different modules in a protein, Pfam (50) and SMART (51, 52) were used.

## Supplementary Material

Supplementary File

## Data Availability

All study data are included in the article and/or *SI Appendix*. The atomic coordinates and structure factors have been deposited in the Protein Data Bank (ID codes 7ZGN and 7ZGM) (53, 54).
