# Supplementary material for: Plant N-glycan breakdown by human gut Bacteroides
Source: Proc Natl Acad Sci U S A. 2022 Sep 19;119(39):e2208168119. doi: 10.1073/pnas.2208168119 (PMC9522356; doi:10.1073/pnas.2208168119)
Supplement: Supplementary File [file pnas.2208168119.sapp.pdf]

PNAS

Supplementary Information for

Plant N-glycan breakdown by human gut *Bacteroides*.

Lucy I. Crouch[1]*, Paulina A. Urbanowicz[2], Arnaud Baslé[3], Zhi-Peng Cai[4], Li Liu[4], Josef Voglmeir[4], Javier M. Melo Diaz[2,5], Samuel T. Benedict[6], Daniel I.R. Spencer[2], and David N. Bolam[3]*

[1]Institute of Microbiology and Infection, College of Medical and Dental Sciences, University of Birmingham, Birmingham, B15 2TT.
[2]Ludger Ltd, Culham Science Centre, Oxfordshire, OX14 3EB, UK.
[3]Biosciences Institute, Newcastle University, Newcastle upon Tyne, NE2 4HH, UK.
[4]Glycomics and Glycan Bioengineering Research Center (GGBRC), College of Food and Technology, Nanjing Agricultural University, Nanjing, China.
[5]Chemistry Department, Royal College of Surgeons in Ireland, 123 St Stephen Green, Dublin 2, Ireland.
[6]School of Biosciences, University of Birmingham, Edgbaston, University of Birmingham, Birmingham, B15 2TT.
* Lucy I. Crouch and David N. Bolam

Email: david.bolam@ncl.ac.uk or l.i.crouch@bham.ac.uk

This PDF file includes:

Supplementary Results and Discussion
Tables S1 to S6
Figures S1 to S14
SI References

Supplemental Results and Discussion

Comparison between the structures of B035DRAFT_03341^{PNGase} and EMTypeII in terms of the α1,3-fucose binding pocket. When the structures of B035DRAFT_03341^{PNGase} and EMTypeII were compared in terms of the α1,3-fucose pocket, the residues forming this pocket were largely identical and in the same position. However, there were two slight differences that might be related to the difference in activity towards N-glycans without α1,3-fucose (Fig. S6).

The first difference was the positioning of an asparagine (N422 in B035DRAFT_03341^{PNGase}) was slightly closer to the where the fucose would bind making the pocket of B035DRAFT_03341^{PNGase} considerably tighter than in EMTypeII and suggesting a more specific accommodation of fucose in the former enzyme (Fig. S6). From the structures, it looks like the reason the loop that this asparagine sits on is slightly higher in B035DRAFT_03341^{PNGase} is due to the presence of an adjacent tyrosine (Y423; compared to a valine in EMTypeII). The larger tyrosine requires more space than the valine within the protein core, so pushes this loop up. It might be that this tighter pocket promotes a requirement for an α1,3-fucose in B035DRAFT_03341^{PNGase} rather than just an accommodation of this sugar in EMTypeII.

The only other difference we could find in these pockets was a cysteine (C563) in B035DRAFT_03341^{PNGase}, whereas there was an asparagine (N558) in the same position in EMTypeII (Fig. S6). The position of these residues is at the deepest part of the pocket. When the surface was compared in this area, it was found to be much deeper for the asparagine compared to the cysteine. This may be another reason the pocket appears less open in B035DRAFT_03341^{PNGase} compared to EMTypeII.

However, without a fucose bound in these structures, these observations are currently only speculative as to the structural basis of the observed differences in activity between B035DRAFT_03341^{PNGase} and EMTypeII.

Removal of the antenna GlcNAc from plant complex N-glycans. In a previous study on the degradation of mammalian biantennary complex N-glycan by *B. thetaiotaomicron*, we characterized a number of GH20 enzymes that had specificity towards the antennary GlcNAcs(1). Three of these GH20 enzymes are in a locus that also encodes the sole GH33 sialidase in the *B. thetaiotaomicron* genome, an esterase that can act on a variety of different acetylated sialic acids, a GH2 galactosidase with specificity towards the LacNAc decorations common to mammalian complex N-glycans, and a GH2 mannosidase with specificity towards the Manβ1,4GlcNac linkage present in all N-glycans (Fig. S14A). The three GH20 enzymes in this locus were characterized to have different, but complementary, specificities towards the GlcNAc's on complex N-glycans. BT0456^{GH20} can remove antennary GlcNAcs, but has a preference towards one arm. BT0459^{GH20} can hydrolyse antennary GlcNAc's, but this activity is hindered by the presence of bisecting GlcNAc. In contrast, BT0460^{GH20} can remove antennary and bisecting GlcNAc's, including removing the bisecting when the antennae still have galactose and sialic acid present. BT0456^{GH20} cannot remove the bisecting if the sialic acid and galactose are still present on the antenna and BT0459 will not remove bisecting. *B. massiliensis* has a syntenic locus to this one with additional putative CAZymes. The three GH20 enzymes in this locus (B035DRAFT_00419^{GH20}, B035DRAFT_00421^{GH20}, and B035DRAFT_00425^{GH20}) have 63, 73, and 73 % identity to BT0460, BT0459, and BT0456, respectively. We expressed and tested these against soya N-glycan that had been treated with B035DRAFT_00996^{GH2} and B035DRAFT_03357^{GH29} to remove the antennary galactose and fucose, respectively. Removal of antennary GlcNAc was observed with B035DRAFT_00421^{GH20} only (Fig. S14B). It may be that the other two GH20 enzymes are sterically hindered by the bisecting β1,2-xylose. These data show that *B. massiliensis* encodes at least one enzyme that is capable of removing the antennary GlcNAc's on plant complex N-glycans.

Comparison between plant N-glycan degradation pathways in *B. massiliensis* and a bacterial phytopathogen. *Xanthomonas campestris* pv. *campestris* causes black rot disease in *Brassica* plant species and in a previous study a set of genes upregulated in the presence of GlcNAc was explored in terms of plant N-glycan degradation(2). These genes were predicted to be putative CAZymes from a range of families and their subsequent characterisation revealed some comparable observations to the work described here. Firstly, a GH92 (NixK) was able to remove the α1,3-mannose from a plant N-glycan heptasacchairde, akin to what was observed here for B035DRAFT_03340^{GH92} (42 % identity between these two enzymes). Furthermore, without the removal of this mannose, the activity of other enzymes was blocked, similar to that observed with the *B. massiliensis* enzymes.

Plant N-glycan β1,2-xylosidase activity was also observed with a GH3 family member (NixI) from *X. campestris*. NixI has a low identity to B035DRAFT_00995^{GH3} of 33 %, but the specificity of acting after the removal of the α1,3-mannose is the same. In terms of core α1,3-fucosidase activity, a GH29 (NixE) could remove this sugar, but only when all mannose sugars had been removed, which is not the case for B035DRAFT_02132^{GH29}. It is worth noting that the substrate used in this study was a glycopeptide produced from trypsin degradation of avidine produced in corn and not a free N-glycan, which may influence the activities observed.

There was no endo-acting enzyme activity characterised for the *X. campestris* system, although interestingly a GH18 was present in the *X. campestris* pv. *campestris* GlcNAc-activated locus. The GH18 is a likely candidate for removal of the N-glycan in *X. campestris*, unlike *B. massiliensis* which employs a PNGase.

Assessing the activity of B035DRAFT_00997^{sulfatase}. Sulfated N-glycans have been observed in a wide variety of organisms ranging from animals to viruses (Fig. S15)(3-6). These decorations can take the form of GalNAc-6S, GalNAc-4S, Gal-3S, Gal-6S, and Man-6S(36). To our knowledge, sulfation of plant N-glycans has not yet been observed, however, with N-glycan sulfation being so widespread throughout other organisms it would be surprising if it was not also present in some plants.

A putative sulfatase adjacent to the genes for the xylosidase and galactosidase (B035DRAFT_00997) was assessed for activity against a variety of sulfated monosaccharides and oligosaccharides (Fig. S15). No activity could be observed against the sulfated substrates tested.

Degradation of high-mannose N-glycan structures by gut *Bacteroides*. The degradation of high mannose N-glycans in *B. thetaiotaomicron* has previously been described(7). This work showed three GH92 enzymes BT3990, BT3991, and BT3994 would hydrolyse the terminal α1,2-, α1,3-, and the first α1,6-mannose from high mannose N-glycans, respectively, to leave a Manα1,6Manβ1,4GlcNac trisaccharide. Homologues of these three enzymes were adjacent to the plant-N-glycan degrading genes in *B. helcogenes*, therefore it appears that this species has the genes required to degrade high-mannose and plant complex N-glycans in the same place in the genome. Homologues of these GH92 enzymes were also traced throughout the other species assessed in this study and found to be well-conserved throughout, including *B. massiliensis* (Fig. S8). Phylogenetic analysis of all the GH92 enzymes from the functional analysis was carried out and these clustered into five groups (Fig S16). Three of these are likely the GH92 enzymes acting high-mannose N-glycans, one group are likely all α1,3-mannosidases that can accommodate β1,2-xylose (homologues of B035DRAFT_03340^{GH92}) and one remains uncharacterised.

Supplemental Materials and Methods

Enzymatic papaya N-glycan release and 2-AB labelling. The applied N-glycan release method is based on the procedures described by Wilson et al. (8) and Du et al. (9). Briefly, papaya (ca. 2 g) were blended in a 2 ml glass homogenizer, transferred into a 2 ml centrifuge tube and then centrifuged (20,000g for 20 min at 4°C). One mL of the clear supernatant was mixed with 1 mL of aqueous trichloroacetic acid (TCA) solution (2 M) and then centrifuged (20,000g for 30 min at 4°C). The resulting pellet was once washed with 1 ml distilled water to remove TCA. The pellet was then re-suspended in 70 µl of distilled water, 28.5 µl of MES buffer (200 mM, pH 7.0) and 12.5 µl of denaturation solution (2% SDS (w/V) and 2-mercaptoethanol (1 M) in water) were added. The mixture was heated at 100°C for 5 min in a heating block. After cooling, 19 µl of a Triton solution (10% w/V) was added followed by the addition of 100 µlL of purified PNGase, and the mixture was incubated overnight at 37°C. The supernatant was collected by centrifugation at 12000 rpm for 20 min and purified with Supelclean ENVI Carb solid-phase extraction (SPE, 500 mg bed volume) columns. The SPE columns was activated with 3 ml of 80% acetonitrile containing 0.1% trifluoroacetic acid (TFA, V/V) and equilibrated with the same volume of distilled water. The sample was loaded to the SPE column and then washed with 3 mL of distilled water. N-glycans were eluted with 20% and 40% acetonitrile containing 0.1% TFA (v/v), collected, dried, and labeled with 2-AB. To do so, an aliquot (10 µl) of 2-AB labelling solution (35 mM of 2-AB and 0.1 M of sodium cyanoborohydride in dimethyl sulfoxide/acetic acid (7:3 v/v)) was added, and the mixture was incubated at 65°C for 4 h.

Papaya N-glycan separation. Chromatographic separation of oligosaccharides was carried using a Nexera UPLC system (Shimadzu Corporation, Kyoto, Japan), consisting of a DGU-20A5R degasser unit, a LC-30AD pump, a SIL-30AC autosampler, and a RF-20Axs fluorescence detector (set at 330 nm excitation and 420 nm emission) adapted from Guo *et* al. (10). Briefly, the analyses were performed using an Acquity BEH Glycan column (Waters 1.7 µm, 2.1 × 150 mm). The mobile phases consisted of NH_4COOH (pH 4.5, 50 mM) in water and acetonitrile for solvents A and B, respectively. The elution methods were set as follows: a linear gradient of 95-78 % B was applied from 0-6 min at a flow rate of 0.5 ml/min; 78-70 % B from 6-20 min at 0.5 ml/min; 70-0 % B in 1 min at 0.25 ml/min; held for 2 min at 0.25 ml/min; 0-95 % B in 2 min at 0.25 ml/min; held for 1.5 min at 0.25 ml/min; the flow rate was then increased from 0.25 to 0.5 mL/min from 26.5-29.5 min; and finally the column was equilibrated with 95 % B for 3.5 min at 0.5 mL/min before the next sample was injected.

MALDI-TOF-MS analysis of papaya samples. UPLC fractions corresponding to selected fluorescence peaks were collected in 2 ml centrifuge tubes, dried using vacuum centrifugation (Speedvac), and re-dissolved in 10 µl of distilled water. 1 µl fractions of this samples were then analysed using a Bruker Auto-flex Speed (Bruker Daltonics, Bremen, Germany) MALDI-TOF-MS spectrometer (equipped with a 1000 Hz Smartbeam laser). Samples were overlaid with 1 µl of 2,5-dihydroxybenzoic acid (DHB) matrix (10 mg/ml DHB in 70% (v/v) aqueous acetonitrile solution). Mass spectra were analysed using the Bruker FlexAnalysis software version 3.3.80, and N-glycan masses were calculated using the GlycoWorkbench software tool (11).

N-Glycan release from soya protein and MALDI analysis. Analytical release of N-glycans from soy proteins was conducted as follows. 1 g of soy protein isolate (purchased from a local supermarket) was washed with deionized (DI) water (3x 10 mL), with centrifugation (10 min X 2500 g) in between each wash. The resulting pellet was homogenized with 20 mL of DI water to form a slurry. 100 µL of the slurry was dried down by vacuum centrifugation and resuspended in 25 µl of 50 mM NaH_2PO_4-Na_2HPO_4 buffer pH 7.5 and boiled for 5 min. Control samples were digested with PNGase F (1 µl, 5 mU, QA-Bio) For B035DRAFT_003341^{PNGase} the final enzyme

concentration was 1 μM. Samples were incubated for 12 h at 37 °C. 100 μl of DI water was added to dilute the sample before For the enzymes assays that included exo-acting fucosidases, galactosidases, and GlcNAc'ases, 25 pmol of procainamide labelled substrate was used. Digestions were carried out in 50 mM sodium phosphate buffer, pH 7, 10 μl for 18 h at 37 °C. Enzymes were used at a final concentration of 1 μl. After incubation, glycans were purified using Ludger clean-up Spin Columns (LC-EXO-A6) and eluted in 200 μl of water. The samples were dried down by vacuum centrifugation, solubilized in 20 μl of water, and analysed by MALDI-MS. MALDI-MS analysis was completed using a Bruker Auto-flex Speed (Bruker Daltonics, Bremen, Germany). The spectrometer was operated in positive ion mode. Spectra were acquired in the mass range 900–3500 m/z at a laser intensity of 50%. The Mass Spectrometry (MS) data were further processed using Flex analysis3.5; sample preparation was as follows 0.5 μl of Super-DHB matrix (50 mg/mL in (50:50 [v/v] H_2O: acetonitrile)), was spotted on a ground steel target, 0.5 μl the sample was added on top and allowed to dry. 20 μl of aliquots of the diluted samples were dried down by vacuum centrifugation and labelled with procainamide prior to UHPLC-MS analysis.

Analysis of mass spectrometry data. Mass spectrometry of procainamide-labelled glycans was analysed using Bruker Compass Data Analysis Software and GlycoWorkbench(11). Glycan compositions were elucidated on the basis of MS^2 fragmentation and previously published data

Isolation of plant complex N-glycans from soya proteins. A total of 20 g of soya protein isolate were processed as follows: 2.5 g of soy protein isolate were placed in 50 mL centrifuge tubes, for a total of eight tubes. To each tube 25 mL,50 mM NaH_2PO_4-Na_2HPO_4 pH 6.0, 0.05 % NaN_3 buffer was added (1:10 solid-liquid ratio), and the samples were denatured by boiling for 5 min at 100 °C. The tubes were allowed to cool down and 60 µL of PNGaseL (2 mg/mL) were added and allowed to incubate for 2d at 37 °C. After release samples were centrifuged (30 min 2500 g). and the pellet was washed thrice with DI water (20 mL), supernatants were combined and concentrated by rotary evaporation to 20 mL. Acetone was added to achieve a concentration of 50% (v/v) and allowed to cool at –20 °C for 1h to precipitate proteins, the supernatant was separated by centrifugation (30 min X 2500 g). The pellet was washed twice with 10 mL of 50% acetone, and the washings were combined with the supernatant and concentrated to dryness using a rotary evaporator. The dry residue was resuspended in 5 mL of H_2O +0.1 % TFA (v/v) and loaded to a 5 g C18 Supelclean^{TM} LC18-SPE cartridge pre-conditioned with 50 mL methanol, followed by 50 mL H_2O + 0.1 % (v/v) TFA to remove residual proteins and other hydrophobic contaminants and washed with 50 mL of H_2O +0.1% TFA, in 5 mL of DI water and loaded to a 2x High prep 26/10 Sephadex-G25 columns. Glycan containing fractions were detected with MALDI-MS and dried down using a SpeedVac vacuum concentrator. Dried glycan samples were resuspended in 70% acetonitrile. Injections of 1.5 mL were applied to a semi-preparative HILIC- column (TSKgel-amide-80, 7.8 i. d x300 mm, 10 µm, Tosoh Biosciences) at 50°C on a Dionex Ultimate 3000 UHPLC with an automatic fraction collector. Elution was performed at a flow rate of 2.0 mL/min. Solvent A was 50 mM ammonium formate (pH 4.4), solvent B was acetonitrile. The column was equilibrated with 70 % solvent B. The gradient elution parameters were 60-48 % solvent B with a linear gradient over 68 min. Detection was carried out at 214 nm and glycan peaks were collected as they eluted from the column, MALDI-MS was used for fraction identification. The fractions containing plant complex N-glycans were combined and concentrated to dryness, salts were removed using a 2x High prep 26/10 Sephadex-G-25 column and subjected to an additional round of purification through the TSKgel-amide-80 column. The fractions containing plant complex N-glycans were combined once again and salts were removed using 2x High prep 26/10 Sephadex-G-25 column.

Exoglycosidase digestion of plant complex glycans from soya proteins.150 pmol of the purified soya protein was labelled with procainamide and used as a substrate for exoglycosidase reactions. For each exoglycosidase digestion, 10 pmol of procainamide labelled soya protein were used.

Digestions were carried out in 50 mM sodium phosphate buffer, pH 7 in a final volume of 10 µl for 18h at 37°C. Exoglycosidases were used at a final concentration of 1 µM. After incubation, glycans were purified using a Ludger LC-EXO-96 plate and eluted in 200 µl of water. The samples were then dried down by vacuum centrifugation, solubilised in 30 µl of water and analysed by UHPLC-MS.

SI References

1. Briliute J*, et al.* (2019) Complex N-glycan breakdown by gut Bacteroides involves an extensive enzymatic apparatus encoded by multiple co-regulated genetic loci. *Nature microbiology* 4(9):1571-1581.
2. Dupoiron S*, et al.* (2015) The N-Glycan cluster from Xanthomonas campestris pv. campestris: a toolbox for sequential plant N-glycan processing. *The Journal of biological chemistry* 290(10):6022-6036.
3. Kim J*, et al.* (2019) N-glycans of bovine submaxillary mucin contain core-fucosylated and sulfated glycans but not sialylated glycans. *International journal of biological macromolecules* 138:1072-1078.
4. Kurz S*, et al.* (2013) Hemocytes and plasma of the eastern oyster (Crassostrea virginica) display a diverse repertoire of sulfated and blood group A-modified N-glycans. *The Journal of biological chemistry* 288(34):24410-24428.
5. Suzuki N, Abe T, & Natsuka S (2022) Structural analysis of N-glycans in chicken trachea and lung reveals potential receptors of chicken influenza viruses. *Scientific reports* 12(1):2081.
6. Hykollari A*, et al.* (2018) Isomeric Separation and Recognition of Anionic and Zwitterionic N-glycans from Royal Jelly Glycoproteins. *Molecular & cellular proteomics : MCP* 17(11):2177-2196.
7. Cuskin F*, et al.* (2015) Human gut Bacteroidetes can utilize yeast mannan through a selfish mechanism. *Nature* 517(7533):165-169.
8. Wilson IB*, et al.* (2001) Analysis of Asn-linked glycans from vegetable foodstuffs: widespread occurrence of Lewis a, core alpha1,3-linked fucose and xylose substitutions. *Glycobiology* 11(4):261-274.
9. Du YM, Zheng SL, Liu L, Voglmeir J, & Yedid G (2018) Analysis of N-glycans from Raphanus sativus Cultivars Using PNGase H. *Journal of visualized experiments : JoVE* (136).
10. Guo RR*, et al.* (2020) Discovery of Highly Active Recombinant PNGase H(+) Variants Through the Rational Exploration of Unstudied Acidobacterial Genomes. *Frontiers in bioengineering and biotechnology* 8:741.
11. Ceroni A*, et al.* (2008) GlycoWorkbench: a tool for the computer-assisted annotation of mass spectra of glycans. *Journal of proteome research* 7(4):1650-1659.

Supplementary Table S1. Percentage identity between putative PNGase enzymes from species of *Bacteroides*.

	Bache_1482	B035DRAFT_03341	BACDOR_01199	BVU_0565	JCM17136DRAFT_02163	BACCOPRO_01102	C510DRAFT_01797	Ga0131163_11613	BACCOPRO_01256	C510DRAFT_02401	B035DRAFT_01659	JCM17136DRAFT_01338	BVU_2763	BACDOR_04367	BACPLE_01865	BACCOP_00373	BF0811	Ga0057464_101111	Ga0052865_00003	Bache_0045	
Bache_1482<br>*B. plebius*	100	78	75	76	77	77	72	33	35	35	34	35	35	35	33	33	35	35	35	35	Group 2
B035DRAFT_03341<br>*B. massiliensis*		100	74	76	77	78	74	34	36	36	34	35	35	35	35	35	36	36	35	36	
BACDOR_01199<br>*B. dorei*			100	97	81	81	73	34	36	36	35	36	36	35	36	36	36	36	37	35	
BVU_0565<br>*B. vulgatus*				100	81	82	74	34	37	36	35	36	36	35	36	36	35	36	37	35	
JCM17136DRAFT_02163<br>*B. sartori*					100	82	74	34	36	36	35	36	36	36	35	35	35	36	35	35	
BACCOPRO_01102<br>*B. coprophilus*						100	88	34	36	36	35	35	35	35	35	37	36	34	35	34	
C510DRAFT_01797<br>*B. barnesiae*							100	32	35	35	34	35	35	35	34	35	37	35	35	33	
Ga0131163_11613<br>*B. luti*								100	55	52	56	54	56	55	56	56	58	58	54	54	Group 1
BACCOPRO_01256<br>*B. coprophilus*									100	73	67	70	71	71	70	67	71	70	58	59	
C510DRAFT_02401<br>*B. barnesiae*										100	68	68	70	70	70	68	68	67	58	59	
B035DRAFT_01659<br>*B. massiliensis*											100	75	77	77	69	66	69	68	60	63	
JCM17136DRAFT_01338<br>*B. sartori*												100	97	97	70	68	72	69	59	60	
BVU_2763<br>*B. vulgatus*													100	99	72	70	74	72	60	60	
BACDOR_04367<br>*B. dorei*														100	72	70	74	71	60	60	
BACPLE_01865<br>*B. plebius*															100	85	73	73	60	59	
BACCOP_00373<br>*B. coprocola*																100	73	72	59	59	
BF0811<br>*B. fragilis*																	100	78	60	59	
Ga0057464_101111<br>*B. neonati*																		100	59	59	
Ga0052865_00003<br>*B. zooleoformans*																			100	65	
Bache_0045<br>*B. helcogenes*																				100	

Supplementary Table S2. Signal peptide predictions of the enzymes characterised in this study.

Species	Locus Tag	Enzyme family	Predicted signal peptide (using SigP5.0)
B. massiliensis	B035DRAFT_03341	PNGase	SPI
B. fragilis	BF0811	PNGase	SPI
B. massiliensis	B035DRAFT_03340	GH92	SPII
B. massiliensis	B035DRAFT_00995	GH3	SPI
B. massiliensis	B035DRAFT_00996	GH2	SPI
B. massiliensis	B035DRAFT_00997	Sulfatase	SPI
B. massiliensis	B035DRAFT_02132	GH29	SPI
B. massiliensis	B035DRAFT_00014	GH29	None
B. massiliensis	B035DRAFT_00409	GH29	SPI
B. massiliensis	B035DRAFT_03357	GH29	SPI

Supplementary Table S3. Data statistics and refinement details.

Data statistics*		
	B035DRAFT_03340	B035DRAFT_03341
Beamline	I04	I03
Date	20/01/17	22/05/17
Wavelength (Å)	0.979	0.979
Resolution (Å)	66.29 – 1.43 (1.45 – 1.43)	45.09 – 1.95 (1.99 – 1.95)
Space group	$P2_12_12_1$	$P2_12_12_1$
Unit-cell parameters		
a (Å)	65.94	59.05
b (Å)	84.35	100.55
c (Å)	132.61	180.37
α = β = γ (°)	90.00	90.00
Unit-cell volume ($Å^3$)	737293	1070942
Solvent content (%)	47	43
No. of measured reflections	959206 (38443)	573096 (31978)
No. of independent reflections	135130 (6477)	79080 (4454)
Completeness (%)	98.8 (97.1)	99.9 (99.6)
Redundancy	7.1 (5.9)	7.2 (7.2)
$CC_{1/2}$ (%)	0.998 (0.502)	0.999 (0.517)
$\langle I\rangle/\langle\sigma(I)\rangle$	9.7 (1.2)	12.6 (1.7)
Refinement statistics*		
Rwork (%)	13.32	18.92
Rfree# (%)	17.22	23.95
No. of non-H atoms		
No. of protein, atoms	5661	4321
No. of solvent atoms	646	336
No. of ion atoms	17	0
R.m.s. deviation from ideal values		
Bond angle (°)	1.22	1.77
Bond length (Å)	0.011	0.011
Average B factor ($Å^2$)		
Protein	20	47
Solvent	33	42
Ions	26	NA
Ramachandran plot+, residues in		
Most favoured regions (%)	97.01	96.1
PDB code	7ZGM	7ZGN

*(Values in parenthesis are for the highest resolution shell).

#5% of the randomly selected reflections excluded from refinement.

+Calculated using MOLPROBITY.

Supplementary Table S4. Percentage identity between putative plant N-glycan specific α1,3-mannosidase GH92 enzymes.

	Ga0057464_101116	BACCOPRO_02654	C510DRAFT_01523	Bache_1481	B035DRAFT_03340	BVU_0555	JCM17136DRAFT_01210	BACDOR_01210
Ga0057464_101116 *B. neonati*	100	73	70	71	72	72	72	72
BACCOPRO_02654 *B. coprophilus*		100	83	78	80	80	80	80
C510DRAFT_01523 *B. barnesiae*			100	77	78	79	78	78
Bache_1481 *B. helcogenes*				100	82	83	82	82
B035DRAFT_03340 *B. massiliensis*					100	87	88	88
BVU_0555 *B. vulgatus*						100	94	95
JCM17136DRAFT_01210 *B. sartori*							100	96
BACDOR_01210 *B. dorei*								100

Supplementary Table S5. Percentage identity between putative plant N-glycan specific β1,2-xylosidase GH3 enzymes.

	C510DRAFT_01523	BACCOPRO_02654	Ga0131163_10288	B035DRAFT_03340	Bache_1482	BVU_0555
C510DRAFT_01523 *B. barnesiae*	100	80	61	65	67	67
BACCOPRO_02654 *B. coprophilus*		100	60	64	66	65
Ga0131163_10288 *B. luti*			100	66	63	62
B035DRAFT_03340 *B. massiliensis*				100	76	75
Bache_1482 *B. plebius*					100	84
BVU_0555 *B. vulgatus*						100

Supplementary Table S6. Percentage identity between putative plant N-glycan specific α1,3-fucosidase GH29 enzymes.

	C510DRAFT_00332	BACCOPRO_02242	JCM17136DRAFT_003055	Bache_1479	B035DRAFT_02132	Bache_1477	C510DRAFT_00333
C510DRAFT_00332 *B. barnesiae*	100	80	74	77	77	69	67
BACCOPRO_02242 *B. coprophilus*		100	73	74	75	67	67
JCM17136DRAFT_003055 *B. sartori*			100	78	79	67	65
Bache_1479 *B. helcogenes*				100	80	70	67
B035DRAFT_02132 *B. massiliensis*					100	71	66
Bache_1477 *B. helcogenes*						100	69
C510DRAFT_00333 *B. barnesiae*							100

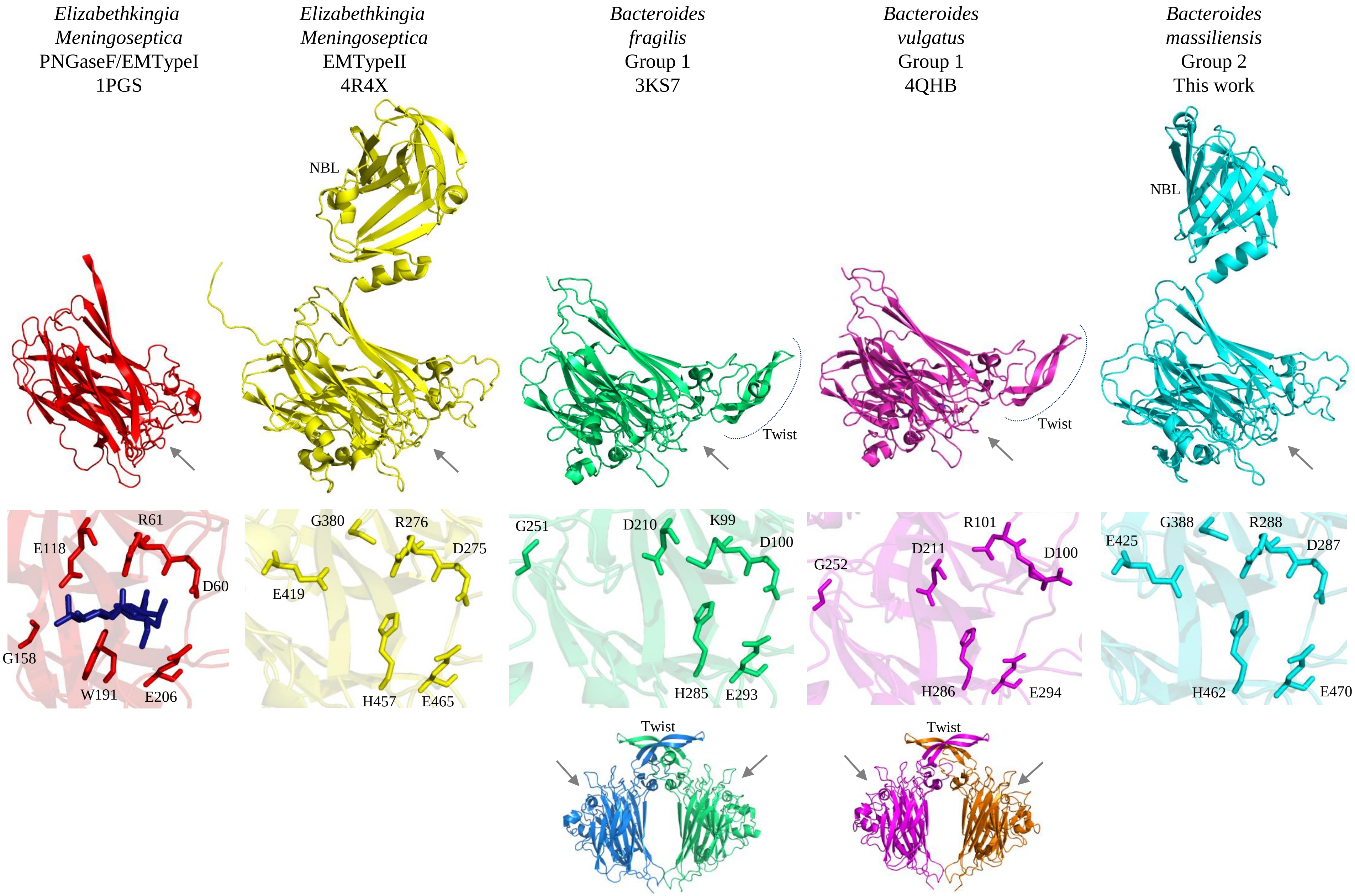

Supplemental Figure S1. Crystal structures of PNGase enzymes from *Elizabethkingia meningoseptica* and *Bacteroides* species. The catalytic domains are lined up and placed in the same orientation for comparison and the grey arrow shows the location of the active site.. The additional N-terminal bowl-like domain from EMTypeII and B035DRAFT_03341^{PNGase} are labelled NBL. The key active site residues from the different enzymes are also shown as sticks. The PNGases from *B. fragilis* and *B. vulgatus* have residues D210 and D211, respectively, that are equivalent to E118 in PNGaseF/EMTypeI. Conversely, B035DRAFT_03341^{PNGase} has G388 and E425, which are equivalent to G380 and E419 in EMTypeII, and form a pocket the likely allows the accommodation of the core α-1,3-fucose found in plant and insect N-glycans. The small β-sheet twist present in the *B. fragilis* and *B. vulgatus* structures is labelled and the way two of these interact is shown.

```
EmenI        ------------------------------------------------------------ 0
Blut11613    ------------------------------------------------------------ 0
Bcopp01256   ------------------------------------------------------------ 0
Bbar02401    ------------------------------------------------------------ 0
Bmas01659    ------------------------------------------------------------ 0
Bsar01338    ------------------------------------------------------------ 0
Bvul2763     ------------------------------------------------------------ 0
Bdor04367    ------------------------------------------------------------ 0
Bple01865    ------------------------------------------------------------ 0
Bcopc00373   ------------------------------------------------------------ 0
Bfrag0811    ------------------------------------------------------------ 0
Bneo101111   ------------------------------------------------------------ 0
Bzoo00003    ------------------------------------------------------------ 0
Bhel0045     ------------------------------------------------------------ 0
EmenII       MLFFLPLLKTNLM--QKILLCSLIT---------GAQMIFAQTYEITYQNSFEGKINPNQ 49
Bhel1482     ------------------MLCILTLSTTISAQNMQKKLKNAKGIEVIYRSVYKGKTIPGQ 42
Bmas03341    -------MRRTGIRNAWLLMLCLLTVSAAAAQNLQKKVKNAKGIEVIYQSSYKGKIRPGQ 53
Bdor01199    -------MKRTILKDVGIGLCFLLSTGTIYAQNYPRKVKNAQGIEVTYQSNYKGRVRPGH 53
Bvul0565     -------MKRTILKNVGIGLCFLLSTGTVCAQNYPGKVKNAQGIEVTYQSNYKGKARPGH 53
Bsar02163    -------MKNFTMKELWLLLCCLMAVSAIHAQNYSKKVKNAKGIEVTYQSSYKGKVRPGY 53
Bcopp01102   ------------------------------------------------------------ 0
Bbar01797    -------MKHLNVKSCCLMVCCLLTVTTAEAENIRKKVKNAPGIEVTYQSSYKGKVAPGQ 53
```

Group 1

Group 2

N-terminal bowl-like domain

```
EmenI        ------------------------------------------------------------ 0
Blut11613    ------------------------------------------------------------ 0
Bcopp01256   ------------------------------------------------------------ 0
Bbar02401    ------------------------------------------------------------ 0
Bmas01659    ------------------------------------------------------------ 0
Bsar01338    ------------------------------------------------------------ 0
Bvul2763     ------------------------------------------------------------ 0
Bdor04367    ------------------------------------------------------------ 0
Bple01865    ------------------------------------------------------------ 0
Bcopc00373   ------------------------------------------------------------ 0
Bfrag0811    ------------------------------------------------------------ 0
Bneo101111   ------------------------------------------------------------ 0
Bzoo00003    ------------------------------------------------------------ 0
Bhel0045     ------------------------------------------------------------ 0
EmenII       NHIISITNSDKTLLFNEKIKNKK-----------ADFPFEVNEINRKNNEVSQFAFLNNN 98
Bhel1482     MQMTVCMD--QVALKNVLPPQEQSPETVGEPTPEIETPVTSNYIDYSSCQAYRLAKLPNG 100
Bmas03341    IKMTVSGN--QVALESVSPKGEK--ETATEGIREDKQPVIKNYIDYAGREAYKWAELPDG 109
Bdor01199    LLMTVSGD--RVSLTNVWSEQND----RPDPRPEDKTPVTGSYIDYTTRQAYRRAELPNG 107
Bvul0565     LLMTVSGD--RVSLTNVWPEQND----RPNPRPEDKTPVTGSYIDYTTRQAYRRAELPNG 107
Bsar02163    LLMTVSTD--RVSLENKRAESKQ--PNDNQVRPEDKTPVTGSYIDYTTCQSYRRAELPNG 109
Bcopp01102   ------------------------------------------------------------ 0
Bbar01797    VLMKVIGD--EVILTSLKPEGKP----AEEIREQDKAPVITNYIDYEACKSYKRAELPDG 107
```

Group 1

Group 2

```
EmenI        ------------------------------------------------------------ 0
Blut11613    ------------------------------------------------------------ 0
Bcopp01256   ------------------------------------------------------------ 0
Bbar02401    ------------------------------------------------------------ 0
Bmas01659    ------------------------------------------------------------ 0
Bsar01338    ------------------------------------------------------------ 0
Bvul2763     ------------------------------------------------------------ 0
Bdor04367    ------------------------------------------------------------ 0
Bple01865    ------------------------------------------------------------ 0
Bcopc00373   ------------------------------------------------------------ 0
Bfrag0811    ------------------------------------------------------------ 0
Bneo101111   ------------------------------------------------------------ 0
Bzoo00003    ------------------------------------------------------------ 0
Bhel0045     ------------------------------------------------------------ 0
EmenII       EIVKTSDNTILAKQEFKPTSETGKILGYNVKKAVTSVNSNTIEVWYTNDLKVKGGPS-IL 157
Bhel1482     KVISAATP-FRIGAGFT-EAGEGKHLGLNCKILRTSLRSNTIEVWYTNDIPFRGTPQANV 158
Bmas03341    KIISAATP-FEFGKGFT-PAGEGKHLGLNCKIARTSINSNTIEVWYTHDIPFRGTPQANV 167
Bdor01199    QVISAVTP-FEFGKGFT-QTGEGKHLGMNCKILRTSINSNTIEVWYTNDIPFRGTPQANV 165
Bvul0565     QVISAVTP-FEFGKGFT-QTGEGKHLGMNCKILRTSINSNTIEVWYTNDIPFRGTPQANV 165
Bsar02163    KIISAMTP-FELGRGFT-EKGEGKHLGLNCKIVRTSINSNTIEVWYTNEIPFRGTPQPNV 167
Bcopp01102   ------------------------------------------------------------ 0
Bbar01797    RIISAATP-FEYGKGFK-EVGTDKVLGLDCKILQTIINSNTIQVWYTTDIPFRGTPQANV 165
```

Group 1

Group 2

```
EmenI         ------------------------------------------------------------ 0     Group 1
Blut11613     ------------------------------MNKNSFLSCGNAFIAMLFLVLAVCGQKLSA 30
Bcopp01256    --------------------------------MWKKVLP---YVAAGVAALSFAACGPKE 25
Bbar02401     --------------------------------MLKKILF---LAAACLFTWTGSAVEHKE 25
Bmas01659     ---------------------------MRRNNLLTFIAS---LALVTSFIMPANAAKHKE 30
Bsar01338     ------------------------------MNLLTFITP---LAVAATFMMPADAANHKE 27
Bvul2763      ------------------------------MNLLTFIAP---LAVAATFMMPADAANHKE 27
Bdor04367     ------------------------------MNLLTFIAP---LAVAATFMMPADAANHKE 27
Bple01865     ----------------------------------MKIVS---MFVSLFVALNVCAAGHKE 23
Bcopc00373    ---------------MIYKNSNFINLKALEFNIDMRTIF---ILFSLLFTLTVNAASHKE 42
Bfrag0811     --------------------------------MNIRLTS---LFVSLFLSVPVWAGGHKN 25
Bneo101111    --------------------------------MNKNLYI---LLIALLSASMAWAGKHKE 25
Bzoo00003     ---------------------------------MNKITC---LLLSLFLLSSALSVSAKK 24
Bhel0045      ------------------------------MLMENKIL-----YLFVVLLGIPGLISARK 25
EmenII        GQDLGLVLKTVRNGSSVVEATSVKKIKALDDQS---------LFNGKNITEKDALTYKDM 208   Group 2
Bhel1482      GVPDGLVLKVIRNGDMVQEASSISPLKKA-EN----------LLPTTWGEALDADDYQYT 207
Bmas03341     GVPDGLVLKVVRNGDMIQEASAITPLKKA-QA----------LLPDSWGEKMDAADYQYT 216
Bdor01199     GVPDGLVLRVVRNGDMIQEATHITPLKK-GKD----------VLPQSWGKSMDAADYQYT 214
Bvul0565      GVPDGLVLRVVRNGDMIQEATHITPLKK-GKD----------VLPQSWGESMDAADYQYT 214
Bsar02163     GIPDGLVLKIVRNGDMVQEAILITPLKKE-AD----------LLPADWGEAMDAADYQYT 216
Bcopp01102    ---------------------------------------------------MDASDFQYT 9
Bbar01797     GVPDGLVLKVVRNGDTVQEAISINPEKETGTS----------LLPTSWGNVMDNADYQYT 215

EmenI         APADNTVNIKTFDKVKNAFGDGLSQS------AEGTFTFPADVTAVKTIK---------M 45    Group 1
Blut11613     SNHNTEFVIKPFNETSVCFNSNDYPDK--VWEGDGLIRLDHGRIVIKKIRVPKFKQNVVV 88
Bcopp01256    YPAQGDCNLTVFQQERVRFCPDSIAN-YTAPDSNGVMRLVNGRILLKKITLPKYQRNIDV 84
Bbar02401     LPAKGDCSLQVFKQERVRFCPDSIGN-ITAPDADGVMRLVNGRILLKKIKLPEYQRNIKV 84
Bmas01659     LPALGDTQIQIFDKTNICFRPDSFAN-YTPASADGVIRLVNGRIILKKISLPDYKRNVRV 89
Bsar01338     LPALGNTHIQVFDKTPVCFRPDSFPN-YTPANADGVIRLVNGRIILKKITLPDYKRDVDV 86
Bvul2763      LPALGNTHIQVFDKTPVCFRPDSFPN-YTPANADGVIRLVNGRIILKKITLPDYKRDVDV 86
Bdor04367     LPALGNTHIQVFDKTPVCFRPDSFPN-YTPANADGVIRLVNGRIILKKITLPDYKRDVDV 86
Bple01865     LPAKGDLNLTVFDKENIHFVPDTYAG-YSTAGADGVIHLVNGRIILKKIQIPDYQRDVTV 82
Bcopc00373    LPAKGNLSLQVFDNANVRFLPNTYPS-FSEADSDGIIHLVNGRIILKKIQIPDYQRDVTV 101
Bfrag0811     LPAKGDLHIPVFENVNVRFSPDTYPDNYNEADGTGVYHLVNGRIILKKITLPEYKRNVSV 85
Bneo101111    LPAKGDQTVRVFEKTNVRFVPGIYPGNYNEADSMGIYHLVNGRIIVKKITLPDYKRNVSV 85
Bzoo00003     HPAMGDLTLRVFDKTPVCFRPDTLKG-YNEPDADGVIRLVNGRIILKKIHLPSYRRNVRV 83
Bhel0045      YPAVGDVNVKVFEKTNVCYRPDRWNG-FNEAGADGVIRLVNGRIILKKIHIPVYKRNVRV 84
EmenII        IWKSRFITIPVFENETINFSDASKSDQ-------VIQRFGNGTIILKKVKIPEIKQGNTI 261   Group 2
Bhel1482      INQSGVITIPVFDQQPICFNGAKLPAT---LEEGIMYPAGGGTIILKKVKLPEYVKNRSI 264
Bmas03341     INQSGVITIPVFDQQTICFNNAKLPDT---LEDGITYSAGGGTLILKKVKLPESAKNRSI 273
Bdor01199     INQSGVITIPVFDQQSICFNNTKLPEV---LKEGVQYSAGGGTILLKKVKLPDYVKNRTV 271
Bvul0565      INQSGVITIPVFDQQSICFNNAKLPEV---LEDGVQYSAGGGTILLKKVKLPDYVKNRTV 271
Bsar02163     INQSGVITIPVFDQQTICFNGAKLPDT---LEEGIQYSAGGGTIILKKIKLPDYVKNRTL 273
Bcopp01102    INQSVVISIPVFSEQRICFNGAKLPEE---VNDQECYSAAGGTIILKNVKLPDYVANRTV 66
Bbar01797     LNQSGVITIPVFNEQTICFNGAKLPDQ---LNDNECYSAAGGTVILKKVKLPEYVANRTV 272
                      :  *.:    :                      .   :*.:          :

EmenI         FIKNECPNKTCDEWDRYANVYVKNK----------------------------------- 70    Group 1
Blut11613     SAK-VKLTSNGDRWDKSGSCFVIPASSVINMIEVA-AGRAKYPAVDSTKLEHFQGIVPGD 146
Bcopp01256    DIK-VELASNGDRWDKSGSVFVLPKESVINLLNIA-EGKQKFPEVDSTKYENMIGIVPGK 142
Bbar02401     MLH-VQVASNGDRWDKSGSVFVLPKNSPINIMSIA-EGKREFPAIDEARLENMKGIVAGP 142
Bmas01659     KLR-LTLASNGDRWDKSGSCFALPKESFVNLMSIA-QGKAAFPPVDSLKYENMIGIVPGK 147
Bsar01338     TLK-VTVASNGDRWDKSGSCFVLPKESVINLMNIA-EGKKAFPAVDSTKYEKMIGIVPGQ 144
Bvul2763      TLK-VTVASNGDRWDKSGSCFVLPKESVINLMNIA-EGKRAFPAVDSTKYEKMIGIVPGQ 144
Bdor04367     TLK-VTVASNGDRWDKSGSCFVLPKESVINLMNIA-EGKRAFPAVDSAKYEKMIGIVPGK 144
Bple01865     SLK-LTVASNGDRWDKSGSCFVLPKNSAVNLLSIA-QGKRKFPEIDSLKLENMIGIVPGK 140
Bcopc00373    SLK-VTVASNGDRWDKSGSCFVLPKNSAVNLLSIA-QGKNKFPEVDSLKLENMVGIIPGK 159
Bfrag0811     SLK-VTLASNGDRWDKSGSCFVLPKSSAINLLTIA-RDGMKFPSVDSLKLEKMVGIVPGK 143
Bneo101111    KLK-LTLASNGDRWDKSGSCFVLPASSAINLLNIA-KGDAKFPKIDSLKLENMNGIIAGK 143
Bzoo00003     AAT-VSVESNGDRWDKTGSCFVLPKESVINMLGVA-RDEQHYPETDTARVELFKGIVSGA 141
Bhel0045      TAT-VTVESNGDRWDKTGSCFVLPRESAVNLLNIA-QGEKRFPAIDSTKLENLKGIIAGD 142
EmenII        FVE-LKQKSNGDAYDRTGDVFIIPQERAISYYTGLTQGVKSLPVYQNGNGKSYQGVALTP 320   Group 2
Bhel1482      FVE-VAQYSDGDAYDRTGSIFVIPTGKKQSFLDAI-RDLKSVPAFQSK-DMAYPALISTP 321
Bmas03341     FVE-VAQYSDGDAYDRTGSVFVIPTDKKQSFLDAI-RNLKSVPSFQAK-DGNYPALISTD 330
Bdor01199     FAE-VVQYSDGDAYDRTGSVFLIPESKRLSFLDAM-RDLKKVPSFHSE-NMDYHGLISTA 328
Bvul0565      FAE-VVQYSDGDAYDRTGSVFLIPEGKQLSFLDAI-RDLKKVPSFRSE-NTDYHGLISTA 328
Bsar02163     FAE-VAQYSDGDAYDRTGSIFMIPTDKAQSFLDAL-RNLKSVPAFQSE-GSDYHGLISTE 330
Bcopp01102    FVE-VAQYSDGDAYDRTGSIFLIPTDKKQSFLDAL-RDLNSVPAFRSG-ETDYHGLVSTD 123
Bbar01797     FVE-VSQYSDGDAYDRTGSVFLIPTDKKQSFLDAL-RNLNSVPSFRSD-STDYHGLVATE 329
                      .  * :*: .. :
```

```
EmenI          TTGEWYEIGRFITPYWVGTEKLPR-------------------GLEIDVTDFKSLLSG-N 110
Blut11613      SFSPNVELMRFMTPFGVGYFSKMDSVTAERRKPVYIDEFAPYAEWEQNITDLYPLLEG-E 205
Bcopp01256     DYLPTVELMRFMTPFGVGHFSAPDDSLSATRRPVYIPHWEKSVTWQQDITQLYPLLEG-E 201
Bbar02401      DYLPTVELMRFMTPFGVGHFSAPDDSLSSKRRPVYIPKWETSVNWVQDITPLYPMLEG-E 201
Bmas01659      DYVPTLELMRFMTPFGVGFYSKKDNEIGAKRKPVYISEWAENVVWEQDITDLYPALEK-E 206
Bsar01338      DYVPTLELMRFMTPFGVGYYSSDNDSLSSKRRPVYIPKWEKSVTWVQDITDLYPALER-E 203
Bvul2763       DYVPTLELMRFMTPFGVGYYSSDNDSLSSKRRPVYIPKWEKSVTWVQDITDLYPALER-E 203
Bdor04367      DYVPTLELMRFMTPFGVGYYSSDNDSLSSKRRPVYIPKWEKSVTWVQDITDLYPALER-E 203
Bple01865      DYLPTIELMRFMTPFGVGHFSEDDDSLSSKRRPVYIPKWEKCVQWEQDITDLYSALKG-E 199
Bcopc00373     NYLPTIELMRFMTPFGVGHYSENDDSLSSKRRPVYIPKWEKCVQWEQDITDLYAALKG-E 218
Bfrag0811      DYLPTVELMRFMTPFGIGHYSNNNDSLSSKRRPVYIPKWESNVTWQQDITDLYPLLEG-E 202
Bneo101111     DYQPTVELMRFMTPFGVGHYSNNEDTLTSKRRPVYIPKWEKQVQWEQDITDLYPLLKG-E 202
Bzoo00003      GYFPAIELMRFMTPFGVGFYSGGDDEKASLRRPVYIDGWAPRAEWTQDVTDRFSSLEG-E 200
Bhel0045       DYLPTLEIMRFMTPFGVGFYSKPDNELSSTRKPVYIDHWEDNVSWTQDVTDRYSALEG-D 201
EmenII         DYLPFIELMRFFTPFGIGHFNEKIQ--------LKGKNWHNNTPYRQDITELRPQLSGKE 372
Bhel1482       YYDTPLELMRFFTAFGVRKFNYN-K--------VKGQDWVDSVLYKSEVTNLAEHLQG-E 371
Bmas03341      DYEAPVELMRFFTGFGVRKFNHN-K--------VKGQHWVDSVIYKSEVTPLASQLQG-E 380
Bdor01199      EYDVPLELMRFFTGFGVRKFNYN-K--------VKGQDWVDSVLYKMEVTPLAEKLEG-E 378
Bvul0565       EYDVPLELMRFFTGFGVRKFNYN-K--------VKGQDWVDSVLYKMEVTPLAEKLEG-E 378
Bsar02163      SYDVPLELMRFFTGFGVRKFNHN-K--------VKGQNWVDSVIYKTEITPLMEKLEG-E 380
Bcopp01102     NYNVPMELMRFFTGFGVRSFNHN-K--------VPGQNWVDSVVYKSEVTPLIERLSG-E 173
Bbar01797      TYDTPIELMRFFTGFGVRSYNHN-K--------VMGQDWVDSVLYKSEVTPLVEHLQG-E 379
                     *: **:* : :   .                          ::*     *.  :
```

Group 1

Group 2

```
EmenI          TELKIYTETWLAKGREYSVDFDIVYGTPDYK---YSAVVPV---VQYNKSSIDGVPYGK- 163
Blut11613      VYVGAFIDTWTKEGYKLSLELDFKESALKCDKLPKRKVVPLVNTVYYY-----GQSIPDL 260
Bcopp01256     AYVGVFIDTWTPEGYVVSMELDIKESKLANDVMPKRHITPLMNTVYYI-----GQTYPDI 256
Bbar02401      AYVGVFIDTWTKEGYLADVRIEVKETEVPCEALPKRQVTPLMNTVYYI-----GQTYPDI 256
Bmas01659      AYIGIFIDTWTAEGYVVGLDIEVKESKISCDALPKRHVQPLINTVYYI-----GQTYPDI 261
Bsar01338      AYVGIYIDTWTAEGYVASMELDVKESKITCDVMPERCVRPLMNTVYYI-----GQTYPDI 258
Bvul2763       AYVGIYIDTWTAEGYVASMELDVKESKITCDVMPERRVKPLMNTVYYI-----GQTYPDI 258
Bdor04367      AYVGIYIDTWTAEGYVASMELDVKESKITCDVMPERRVKPLMNTVYYI-----GQTYPDI 258
Bple01865      VYVGIFIDTWTKEGYVASMELKIKETPVTCEKLVRRHVEPLMNTVYYI-----GQSYPDI 254
Bcopc00373     VYVGIFIDTWTAEGYIASMELNIKETPIACEKLIRRHVEPLMNTVYYI-----GQSYPDI 273
Bfrag0811      AYVGIYIDTWTSEGYLVNADIDVKESRLACDVLPKRHVEPLMNTVYYM-----GQSYPDI 257
Bneo101111     AYVGVFIDTWTPEGYVVSMELDVKESKITCNPLPKRHVEPLMNTVYYI-----GQSYPDI 257
Bzoo00003      AYVGIFIDTWTAEGYVASLTLEVKESAIPEDALLRTRVLPLINTVPYV-----GQSLPDL 255
Bhel0045       VYVGVYIDSWTAEGYLVSLELKVKESDIPEDKLRQTHVLPLVNTVPYQ-----GQNIPDI 256
EmenII         ILIGAFIGNYDKGGHQISLELSIHPD--QQKIVNNNFVLPVFNTTNVMEM--AGQDYPTM 428
Bhel1482       AWIGAYIGNWDAKGHRLSLNLKYYPD--DEHRILK--TIPLFNTVNYLEQ--AGQAYPTF 425
Bmas03341      VWIGAYIGNWDAKGHRLSLKLKYYPD--DERRVNK--AMPLFNTVNYLEQ--AGQAYPVF 434
Bdor01199      AWIGAYIGNWDAKGHRLSLKLKYYPD--EEHRVYN--TLPLFNTVNYLEQ--AGQPYPIF 432
Bvul0565       AWIGAYIGNWDAKGHRLSLKLKYYPD--EEHRVYN--TLPLFNTVNYLEQ--AGQPYPIF 432
Bsar02163      AWIGAYIGNWDAKGHRLSLKLKYYPD--EEHRVYN--AIPLFNTVNYLEQ--AGQPYPIF 434
Bcopp01102     AWIGAYIGNWDAKGHRLSLKLKYYPD--EEHRVYK--SIPLFNTVNYMEQ--AGQPYPIF 227
Bbar01797      AWIGAYIGNWDAKGHRLSLKLKYYPD--DEHRIYK--SIPLFNTVNYMEQ--AGQPYPIF 433
                 :  :  .:   *   .  :.                 *:   .        *
```

Group 1

Group 2

```
EmenI          -AHTLALKKNIQLPTN---TEKAYLRTTISGWGHAKPYDAGSRGCAEWCFRTHTIAINNS 219
Blut11613      FAR-KSLSVPFTLPKNAKNVRLNYITTGHGGHDGGDEF----------VKKENIVSIDGH 309
Bcopp01256     FAR-RAVTTDFTLPRDARNVELKYIVTGHGGHSGGDEF----------VQKQNIVSVDGK 305
Bbar02401      FAR-KPVETTFTLPREAKNVQLKYIVTGHGGHSGGDEF----------VERQNIVSVDGK 305
Bmas01659      FAR-KDVAMDFELPRHAKNVRLKYIVTGHGGHSGGDEF----------VKKRNIVSVDGE 310
Bsar01338      FSR-KDVVMDFDMPEAARNVRLKYIVTGHGGHSGGDEF----------VEKRNIVSVDGK 307
Bvul2763       FSR-KDVVMDFDMPKAAKNVRLKYIVTGHGGHSGGDEF----------VEKRNIVSVDGK 307
Bdor04367      FSR-KDVVMDFDMSKAAKNVRLKYIVTGHGGHSGGDEF----------VEKRNIVSVDGK 307
Bple01865      FAR-KSVSTDFLLPKNAKNVRLKYIVTGHGGHSGGDEF----------VQKRNILSVDGK 303
Bcopc00373     FAR-KSVSADFVLPKNAKNVRLKYIVTGHGGHSGGDEF----------VQKKNILSVDGK 322
Bfrag0811      FAR-RDVSTDFTVPKGAKNIRLKYIVTGHGGHSGGDEF----------VQKRNIISVDGK 306
Bneo101111     FSR-KDVSTEFTVPKGAKNVRLKYVVTGHGGHSGGDEF----------VEKRNILSIDGR 306
Bzoo00003      FAR-RSVTVDAEVPAKAKNLRLQYIATGHGGHSGGDEF----------TQQLNIVRVDGD 304
Bhel0045       FAR-KAVEVPFHLPASARNVRLKYITTGHGGHSGGDEF----------TQQRNLVKVDGE 305
EmenII         FNSDKGVEVEFILTKDLKNAQLRYITTGHGGWGAGDEF----------VPKENSIYLDGK 478
Bhel1482       LGN-DTLRVKFTLNEPVTNARLFYLTTGHGGWGGGDEF----------NQKPNTILLDGQ 474
Bmas03341      FLN-DSLRVRFTLKEPAKNARLFYLTTGHGGWGNGDEF----------NQKPNTVYLDGK 483
Bdor01199      MRQ-DSLIVKFTLKEPAKNARLYYLTTGHGGWGGGDEF----------NQKPNTLYLDGE 481
Bvul0565       MRQ-DSLTVKFTLKEPAKNARLYYLTTGHGGWGGGDEF----------NQKPNTLYLDGE 481
Bsar02163      MRN-DSLTVRFTLKEPVKNARLYYLTTGHGGWGGGDEF----------NQKTNTIYLDGQ 483
Bcopp01102     MLN-DSLKATFTLKEPVKNAKLYYVTTGHGGWGNGDEF----------NQKPNTIYLDGE 276
Bbar01797      MLK-DSLKATFTLKEPVKDAKLYYFTTGHGGWGNGDEF----------NQKPNTIYLDGQ 482
                     :     :       .  *. *  .* . .. :            : : : ::.
```

Group 1

Group 2

```
                                Extra twist region
                                ------------------
EmenI          NTFQHQLGALGCSANPINNQSPGNW---------------------------TPDRAGWC 252
Blut11613      EVIRFIPWRNDCASFRRFNPSTGVWLQKRTASYISEEGKRAEKEIEEPIASSDLSRSNWC 369
Bcopp01256     EALNFIPWRDDCASFRRFNPGTGVWLVKRLASYIDGEGY-AMKEVEEPLGSSDLSRSNWC 364
Bbar02401      TVLDFIPWRDDCASFRRFNPATGVWLIKREAAYIGENGY-EVKEVEEPLASSDLSRSNWC 364
Bmas01659      NVLDFIPWRDDCASFRRFNPGTGVWLIKRLSSYIGKNGY-EEKEIEEPLGSSDLSRSNWC 369
Bsar01338      EVLNFIPWRDDCASFRRFNPATGVWLIPRVAAYIGDKGY-TTKEIEEPLASSDLSRSNWC 366
Bvul2763       EVLNFIPWRDDCASFRRFNPATGVWLIPRVAAYIGDKGY-TTKEIEEPLASSDLSRSNWC 366
Bdor04367      EVLNFIPWRDDCASFRRFNPATGVWLIPRVAAYIGDKGY-TTKEIEEPLASSDLSRSNWC 366
Bple01865      EVYSFVPWRDDCASFRRFNPATGVWLIKRLAAYISEDGY-KTKEVEEPLASSDLSRSNWC 362
Bcopc00373     EVYSFIPWRDDCASFRRFNPATGVWLIERLAAYISEDGY-KTKMVEEPLASSDLSRSNWC 381
Bfrag0811      EVLNFIPWRDDCASFRRFNPATGVWLIKRLASYIGEKGY-TEKEVEEPLASSDLSRSNWC 365
Bneo101111     EVMNFIPWRDDCASFRRFNPATGVWLIKRKASYIGEKGY-AEKEVEEPLASSDLSRSNWC 365
Bzoo00003      TVIHFVPWRTDCASFRRFNPTSGVWLVKRKAAYIGEKGY-ETKEIEEALASSDLSRSNWC 363
Bhel0045       TVLDFIPWRTDCASFRRFNPGSGVWLEKRMASFIGKEGY-EMKEIEEPVASSDFSRSNWC 364
EmenII         LAHAFTPWRTDCGSYRLFNPASGNF--------------------EDGLSSSDLSRSNWC 518
Bhel1482       KIITFIPWRDDCGTYRNLNPCSGNF--------------------SNGLSSSDLSRSNWC 514
Bmas03341      KVISFIPWRDDCGTYRNSNPCSGNF--------------------SNGLSSSDLSRSNWC 523
Bdor01199      KVISFVPWRDDCGTYRNWNPCSGNF--------------------SNGLSSSDLSRSNWC 521
Bvul0565       KVISFVPWRDDCGTYRNWNPCSGNF--------------------SNGLSSSDLSRSNWC 521
Bsar02163      KIISFIPWRDDCGTYRNWNPCSGNF--------------------SNGLSSSDLSRSNWC 523
Bcopp01102     KVITFIPWRDDCGTYRNWNPCSGSF--------------------SNGLSSSDLSRSNWC 316
Bbar01797      KVITFIPWRDDCGTYRNWNPCSGNF--------------------SNGLSSSDLSRSNWC 522
                   .     .*.:    *   * :                             .*:.**

EmenI          PGMAVPTRIDVLNNSLIGSTFSYEYKFQNWTNNGTNGDAFYAISSFVIAKSNTPISAPVV 312
Blut11613      PGSVVAPYSVLLENLSPGD-HSLNIAIPKAQASEGDKMNHWLVSAYIVWDE--------- 419
Bcopp01256     PGSDVVPETAALGDLKAGT-HTFTVSIPEAQPVKGNELNHWLVSAYLVWEE--------- 414
Bbar02401      PGSDVLPETVELGTLSAGE-HTFKVDIPEAEPVDGDKLNHWLVSAYLVWE---------- 413
Bmas01659      PGSDVVPEEVMLGDLKAGK-HTFKISIPEAQEVDGDKLNHWLISAYLVWDE--------- 419
Bsar01338      PGSDVMPEEAVIGDLAAGK-HSFKVSIPEAQQVMAIN----------------------- 402
Bvul2763       PGSDVMPEEAVIGDLSAGK-HSFKVSIPEAQQVDGNKLNHWLVSAYLVWEE--------- 416
Bdor04367      PGSDVMPEEAVIGDLPAGK-HSFKVSIPEAQQVDGNKLNHWLVSAYLVWEE--------- 416
Bple01865      PGSDVLPEVIELPGLQAGK-HTFTVSIPEAQPVNKDELNHWLVSAYLVWEE--------- 412
Bcopc00373     PGSDVVPEEIPLKELQAGK-HTFTVSIPDAQPGNGEELNHWLVSAYLVWDE--------- 431
Bfrag0811      PGSDVVPEEAVIGTLAPGK-HTFTVSIPEAQAVDGNKLNHWLVSAYLVWEE--------- 415
Bneo101111     PGSDVWPEEVALGTLSPGK-HTFSVSIPEAQKIKGNELNHWLVSAYLVWEE--------- 415
Bzoo00003      PGSDVWPVAACLKHLKPGS-HIFAFSIPNAQPAKGEELNHWLISAYLVWEE--------- 413
Bhel0045       PGTDVWPEEVNLSNLVSGS-HILSISVPDAQPADGDKMNHWLISAYLVWDD--------- 414
EmenII         PGTITNPVYINLGNLNAGK-HTIQVKIPQGAPE-GSSQSFWNVSGVLLGQE--------- 567
Bhel1482       PGTVTNPEYICLGNLEAGE-HTLSVQIPQGAPE-GNSNSYWCISGTLIY----------- 561
Bmas03341      PGTVTTPEYIYLGDLEAGE-HTLSVRIPQGAPE-GGSNSYWCISGTLLY----------- 570
Bdor01199      PGTVTNPEYIYLGDLEAGE-HSITVKIPQGAPE-GESNSYWCISGTLIY----------- 568
Bvul0565       PGTVTNPEYIYLGDLEAGE-HSITVKIPQGAPE-GGSNSYWCISGTLIY----------- 568
Bsar02163      PGTVTNPEYIYLGDMEAGE-HILSVQIPQGAPE-GGSNSYWCISGTLIF----------- 570
Bcopp01102     PGTVTNPEYIYLGDLDAGT-HTITVKIPQGAPE-GGSNSYWCISGTLIY----------- 363
Bbar01797      PGTVTNPEYIYLGDLEAGT-HTITVQIPQGAPE-GGSNSYWCISGTLIY----------- 569
               **  .      :     *  .     . .

EmenI          TN    314
Blut11613      --    419
Bcopp01256     --    414
Bbar02401      --    413
Bmas01659      --    419
Bsar01338      --    402
Bvul2763       --    416
Bdor04367      --    416
Bple01865      --    412
Bcopc00373     --    431
Bfrag0811      --    415
Bneo101111     --    415
Bzoo00003      --    413
Bhel0045       --    414
EmenII         --    567
Bhel1482       --    561
Bmas03341      --    570
Bdor01199      --    568
Bvul0565       --    568
Bsar02163      --    570
Bcopp01102     --    363
Bbar01797      --    569
```

Group 1: EmenI – Bhel0045; Group 2: EmenII – Bbar01797

Supplemental Figure S2. Sequence alignment of two PNGases from *Elizabethkingia meningoseptica* and twenty PNGases from thirteen *Bacteroides* species. A sequence alignment to show key differences between the different PNGases. The N-terminal bowl-like domain found in Group 2 PNGases is highlighted in purple and the extra twist region present in the Group 1 PNGases from *Bacteroides* species. The residues that are key to the specificity of accommodating the α1,3-fucose typical of plant N-glycans are highlighted in the same way as in Fig. 1. The residue blocking the α1,3-fucose in the Group I PNGases is highlighted in blue (E118 in PNGaseF/EMTypeI), the glycine replacing this residue in the Group 2 PNGases is highlighted in pink (G380 in EMTypeII), and the glutamic acid replacing the function of E118 is highlighted in green(E419 in EMTypeII). Alignments were carried out using Clustal Omega (see Methods). EmenI is PNGaseF/EMTypeI from *Elizabethkingia meningoseptica*, EmenII is EMTypeII from *Elizabethkingia meningoseptica*, Blut is *Bacteroides luti* DSM 26991, Bcopp is *Bacteroides coprophilus* DSM 18228, Bbar is *Bacteroides barnesiae* DSM 18169, Bmass is *Bacteroides massiliensis* DSM 17679, Bsar is *Bacteroides sartorii* DSM 21941, Bvul is *Bacteroides vulgatus* ATCC8482, Bdor is *Bacteroides dorei* DSM 17855, Bple is *Bacteroides plebius* DSM 17135, Bcopc is *Bacteroides coprocola* DSM 17136, Bfrag is *Bacteroides fraglis* NCTC9343, Bneo is *Bacteroides neonati* M24, Bzoo is *Bacteroides zoogleoformans* ATCC 33285, and Bhel in *Bacteroides helcogenes* DSM 20613.

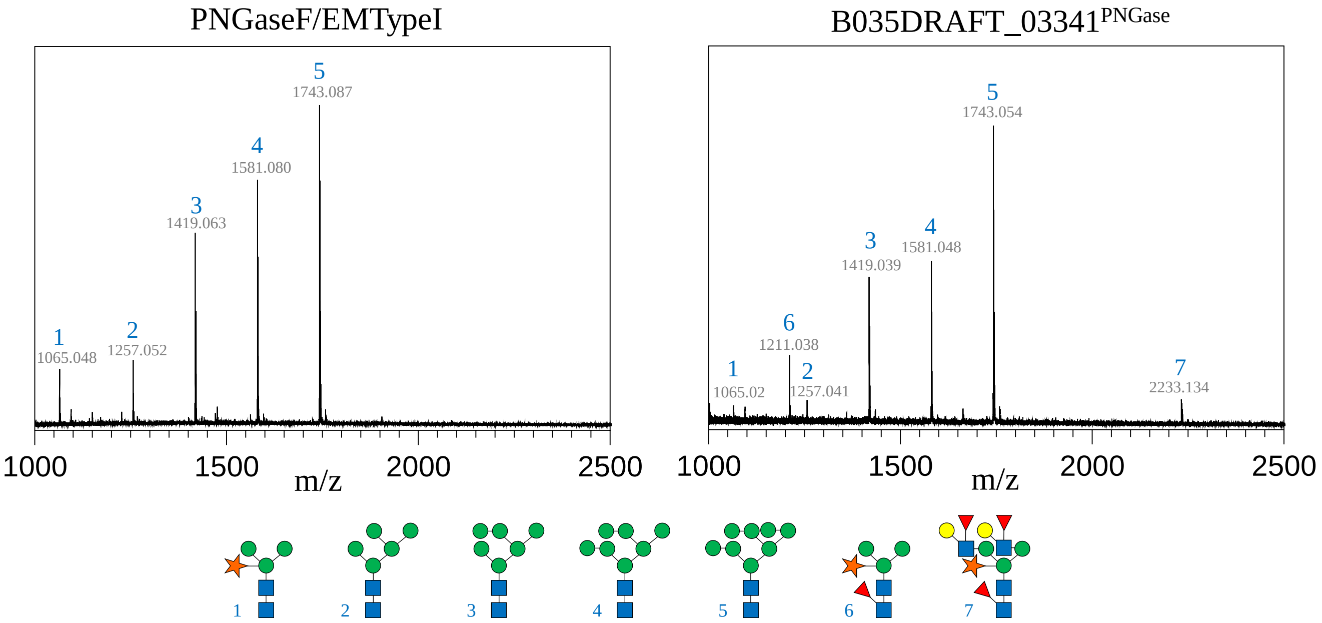

Supplemental Figure S3. MALDI mass spectra of PNGase activity against soya protein extract. These data correspond to the samples from Fig. 2E. Prior to labelling, the finished assay was spotted on to a ground steel target on top of Super-DHB matrix. Data was collected using a Bruker Auto-flex Speed in positive ion mode, range 900-3500 m/z at a 50 % laser intensity. Data was processed using Flex analysis 3.5. The data show the presence of plant-type N-glycans 6 and 7 for B035DRAFT_03341^{PNGase}, but not with PNGaseF/EMTypeII.

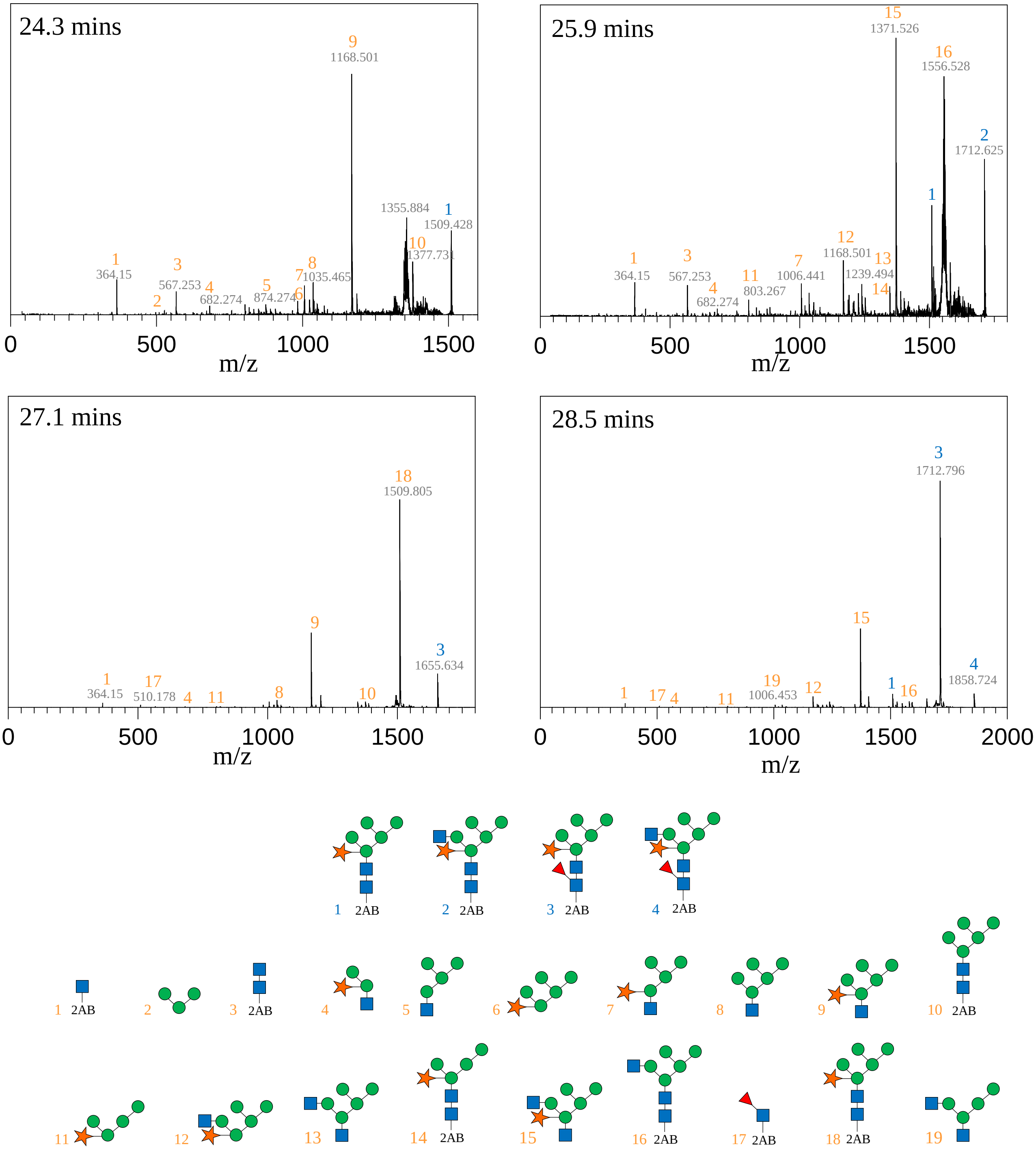

Supplemental Figure S4. MALDI mass spectra of PNGase activity against papaya protein extract. These data correspond to the samples from Fig. 2F. The glycans released by B035DRAFT_03341PNGase and labelled with 2AB as described in Materials and Methods. The numbers shown in blue and orange correspond to the full glycan structures and the fragments, respectively.

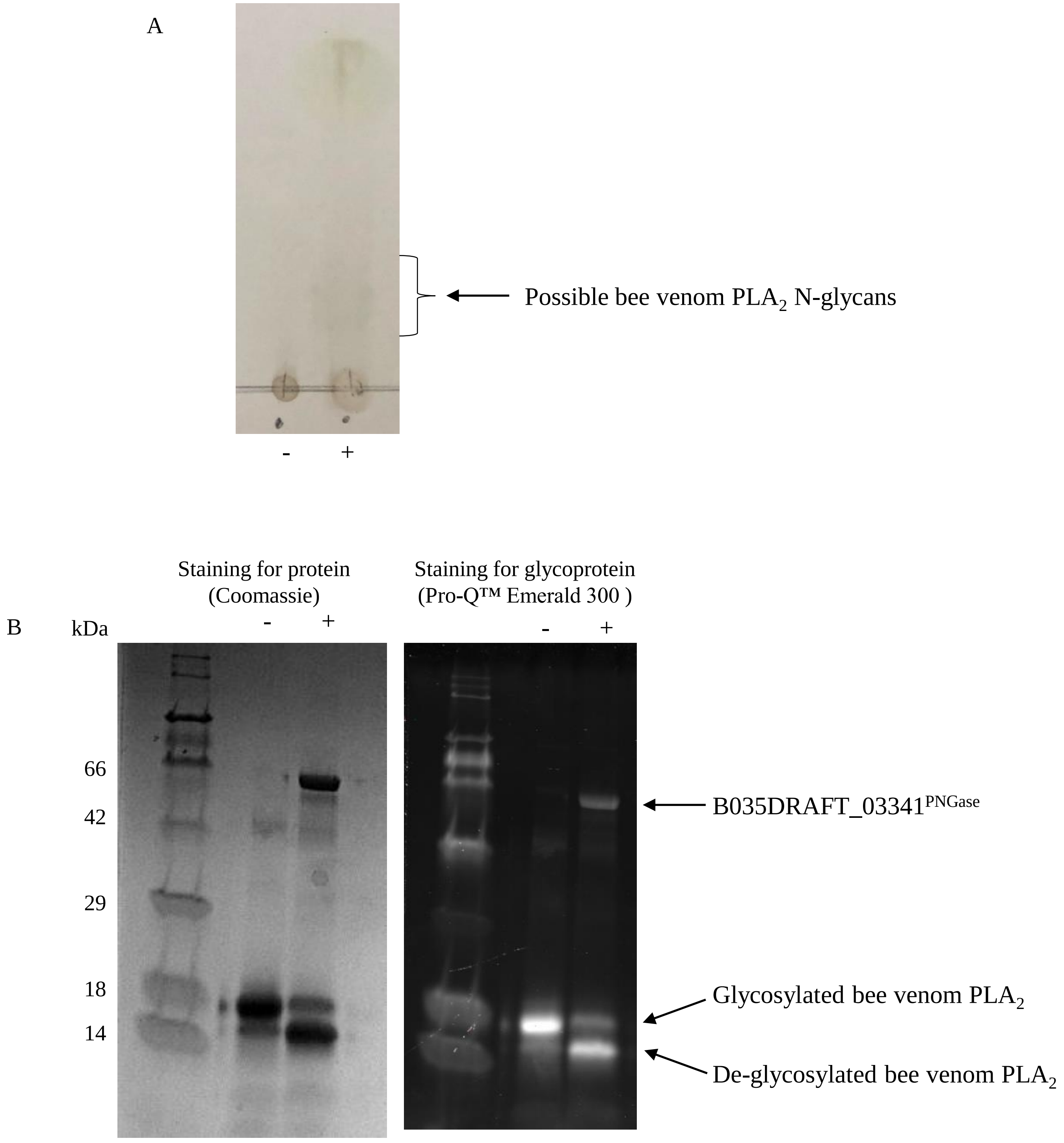

Supplemental Figure S5. Activity of B035DRAFT_03341^{PNGase} against an insect glycoprotein. (A) B035DRAFT_03341^{PNGase} was incubated with phospholipase A_2 from bee venom to determine if insect N-glycans are also a substrate for this enzyme. Assays contained 1 µM enzyme, 0.5 mg/ml substrate, and 20 mM MOPS pH 7 and were carried out overnight at 37 °C. 12 µl of the assay was spotted onto the TLC plate. (B) The same assay was assessed using SDS-PAGE gels either stained for protein or glycoprotein, where 4 µg of PLA_2 was loaded in each lane. A decrease in molecular weight can be seen from the PLA_2 in the presence of B035DRAFT_03341^{PNGase} and there is also reduction in glycoprotein staining for this band compared to the control.

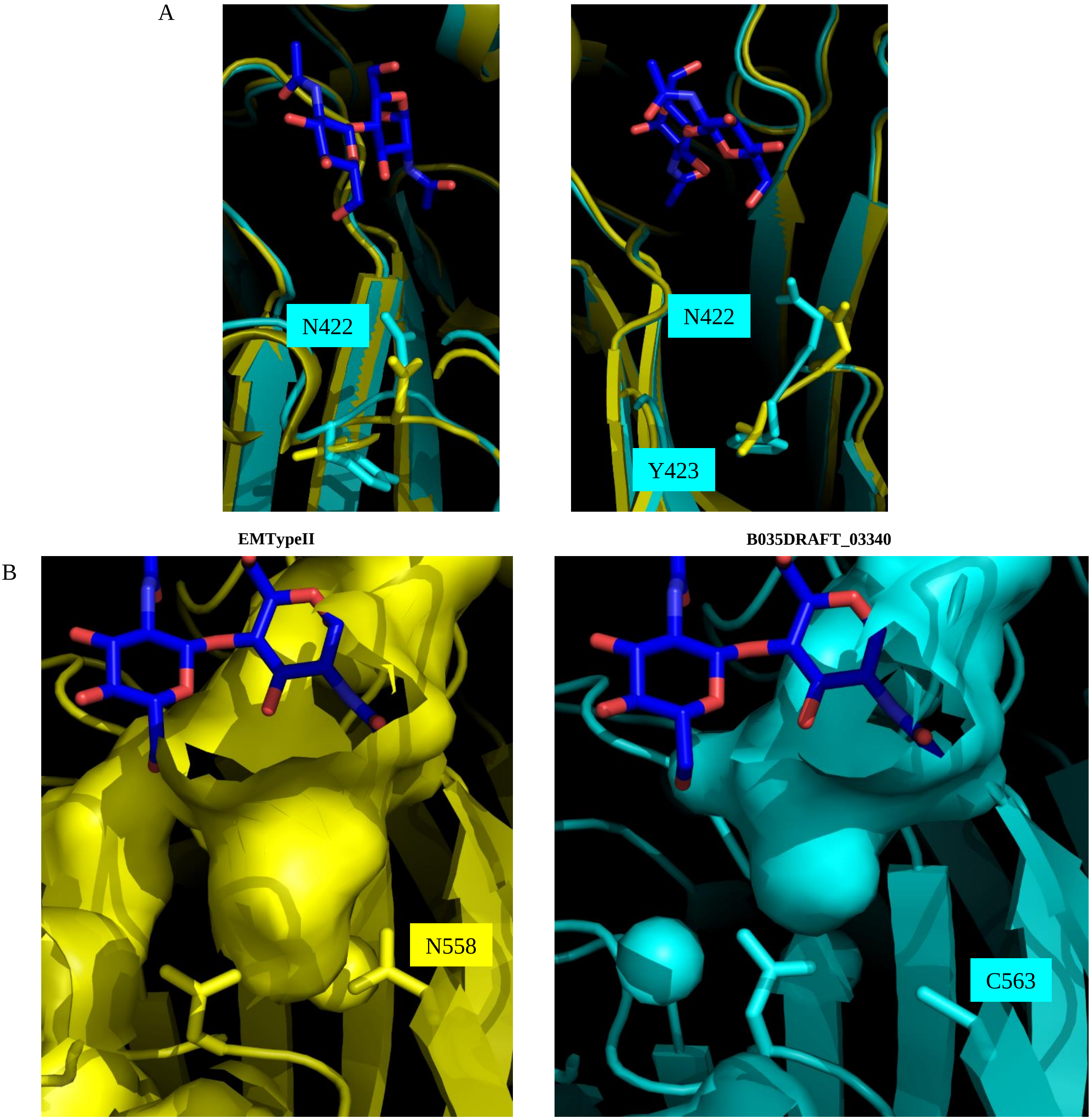

Supplemental Figure S6. A comparison between the α1,3-fucose binding pockets of B035DRAFT_03341^{PNGase} and EMTypeII. (A) The structures of B035DRAFT_03341^{PNGase} and EMTypeII, cyan and yellow respectively, were overlaid with the chitobiose from the EMTypeI (1PNF) structure to compare the residues that are likely involved in the α1,3-fucose binding pockets. One of the differences was the different positioning of an aspargine, which is elevated in the B035DRAFT_03341^{PNGase} structure towards the place where a fucose would bind relative to the EMTypeII structure. Two different views are shown. (B) The second difference between these two structures was one amino acid difference from an asparagine in EMTypeII to a cysteine in B035DRAFT_03341^{PNGase}. In this figure, the surface of the pockets and cavities has been shown to visualise the difference in the α1,3-fucose binding pockets. EMTypeII has a notably deeper pocket than B035DRAFT_03341^{PNGase}, with the asparagine and cysteine residues, respectively, at the bases of these pockets.

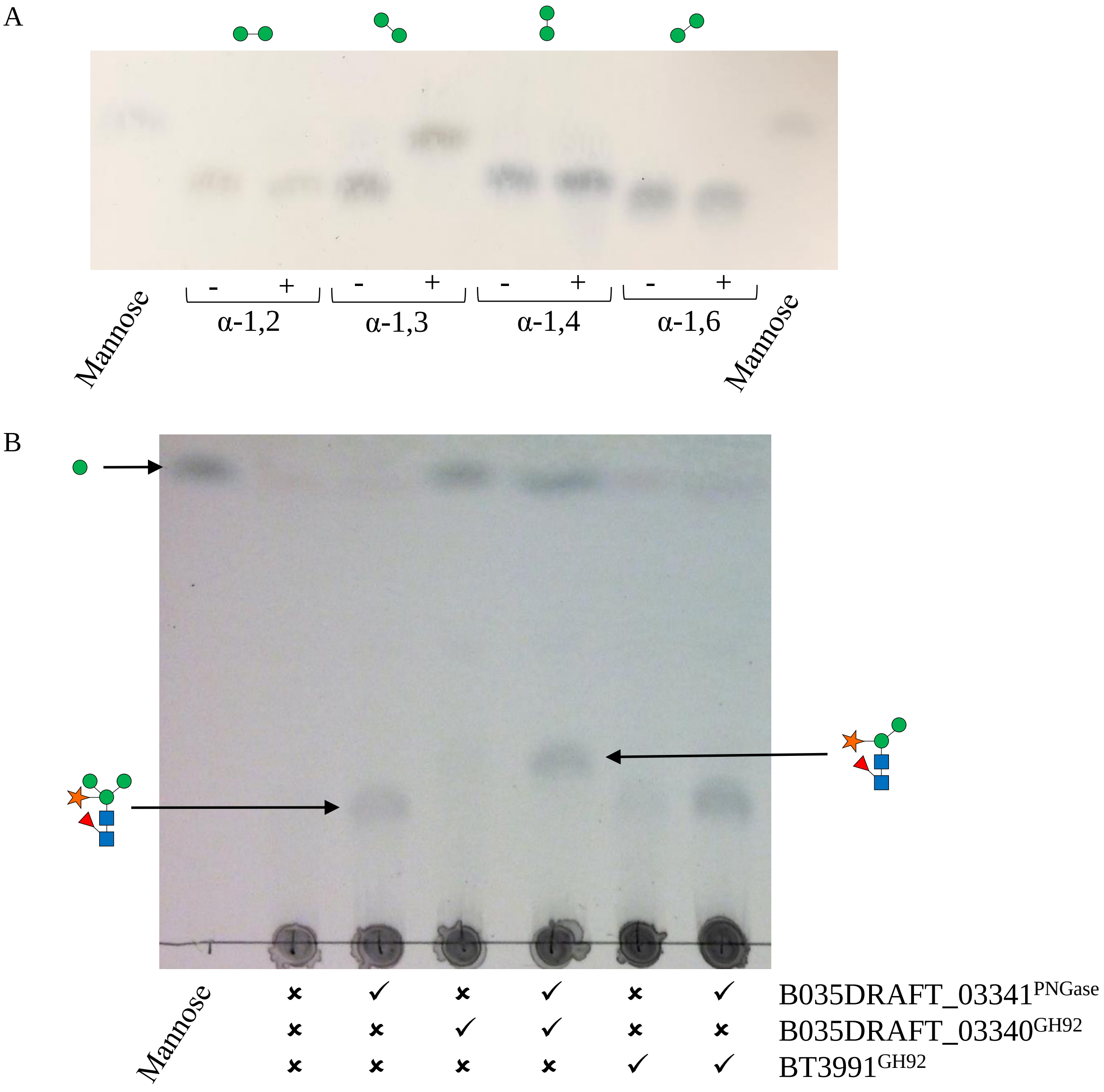

Supplemental Figure S7. Activity of B035DRAFT_03340^{GH92}. (A) B035DRAFT_03340^{GH92} α-mannosidase was incubated with mannobiose with different linkages to determine specificity. Assays contained 1 µM enzyme, 1 mM substrate, and 20 mM MOPS pH 7 and were carried out overnight at 37 °C. 3 µl of the assay was spotted onto the TLC plate. (B) B035DRAFT_03340^{GH92} was incubated against horseradish peroxidase in different combinations with B035DRAFT_03341^{PNGase} and BT3991^{GH92} α-mannosidase. Assays contains 10 mg/ml of glycoprotein, and 20 mM MOPS pH 7 and were carried out overnight at 37 °C. 9 µl of the assay in total (3x 3 µl) was spotted onto the TLC plate.

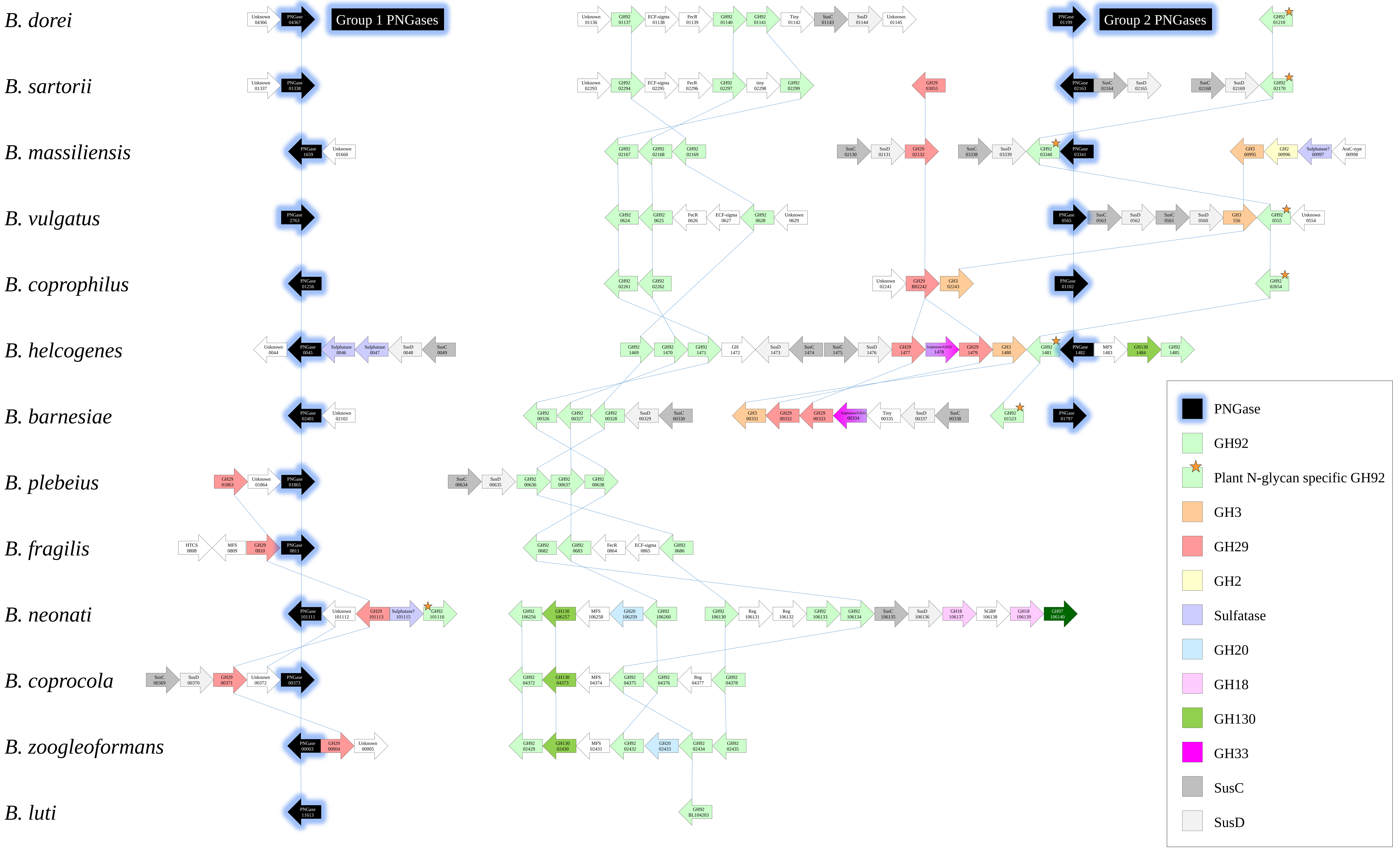

Supplementary Fig. S8. Functional association analysis of genes associated with PNGase genes in *Bacteroides* species. The genes in the same loci as the PNGase genes were explored and homologues were identified in the thirteen *Bacteroides* species. The prefix for the locus tags have been left out for brevity, but are as follows: *B. dorei*, *B. sartorii*, *B. massiliensis*, *B. vulgatus*, *B. coprophilus*, *B. helcogenes*, *B. barnesiae*, *B. plebius*, *B. fragilis*, *B. neonati*, *B. coprocola*, *B. zoogleoformans*, and *B. luti* is BACDOR_, JCM17136DRAFT_, B035DRAFT_, BVU_, BACCOPRO_, Bache_, C510DRAFT_, BACPLE_, BF, Ga0057464_, BACCOP_, Ga0052865_, and Ga0131163_, respectively. The blue lines connect the homologues. Abbreviations include: MFS – major facilitator superfamily, Reg - regulation.

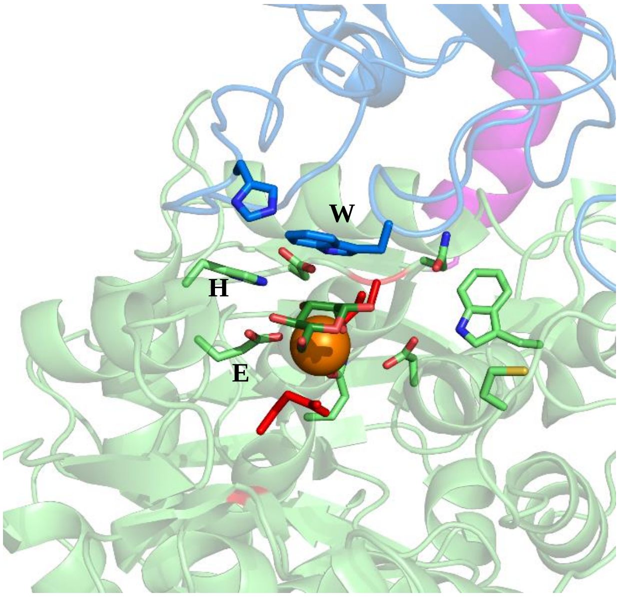

Streptococcus pneumoniae
SP2145 5SW1

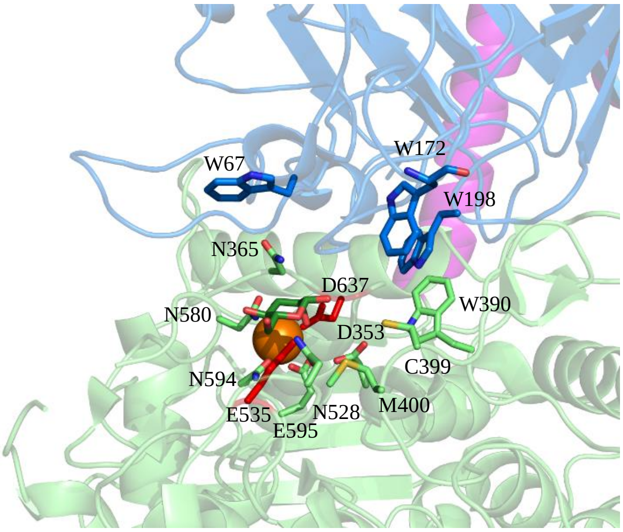

Bacteroides thetaiotaomicron
BT3130 6F8Z

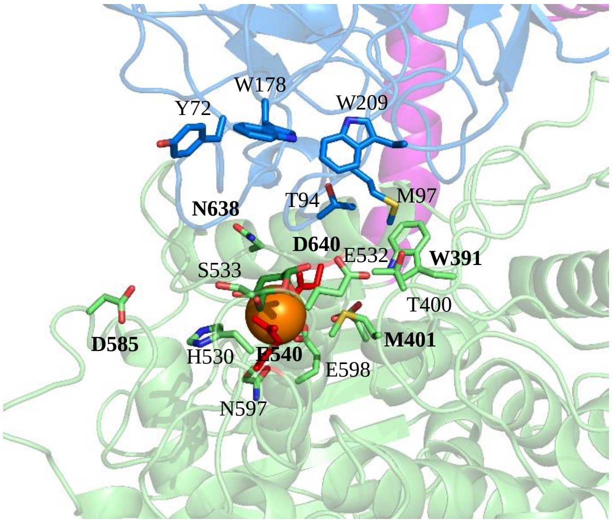

Bacteroides massiliensis
B035DRAFT_03340 7ZGM

Supplemental Figure S9. Comparison of the active sites of an α1,2-mannosidase with the two α1,3-mannosidases. For all structures, the N-terminal β-sandwich domain, the two connecting helices, and the C-terminal $(\alpha/\alpha)_6$-barrel are shown in marine, magenta, and lime, respectively. The metal ion is shown in orange and catalytic residues are shown in red. The structure of the α1,2-mannosidase from *Streptococcus pneumoniae* was crystallised with mannose in the +1 subsite (forest green). The three amino acids which drive the specificity for the α1,2-linkage are highlighted (E, H, and W) and form a lid that sits on top of the substrate. The two available crystal structures for α1,3-mannosidases are also shown (6F8Z and 7ZGM) with the +1 mannose from 5SW1 overlaid into the active site. The residues likely to be interacting with the sugar in the +1 subsite are shown as sticks and labelled. Interestingly, for both these α1,3-mannosidases, there are also aromatic residues contributed from the N-terminal domain that may also act as a “lid” sitting on top of the substrate.

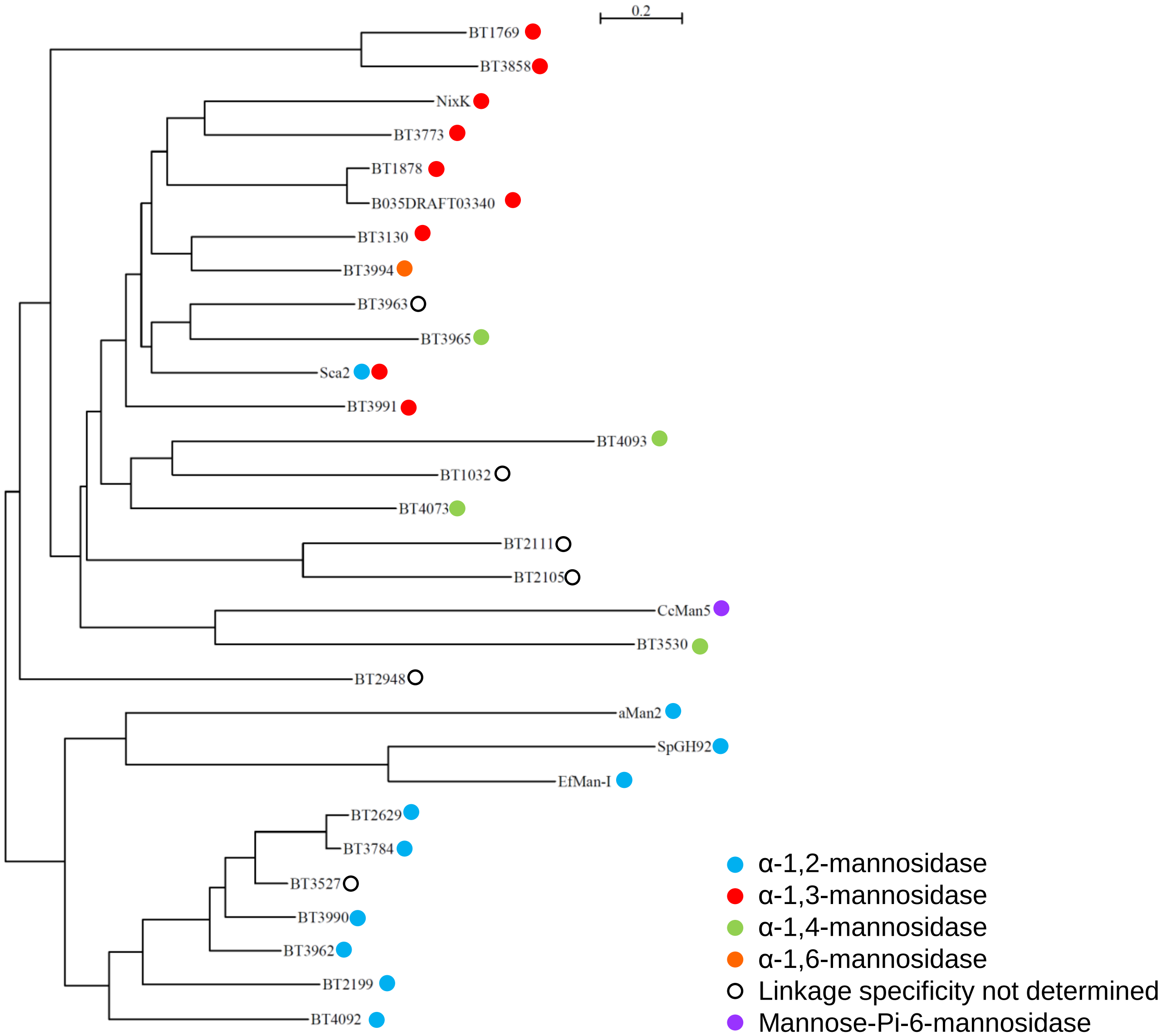

Supplemental Figure S10. Phylogenetic tree of the characterised enzymes from the GH92 family. The sequences of GH92 enzymes that have been characterised in the CAZy database were compared as described in Materials and Methods. Enzymes with similar specificities can be seen to be grouping together although it should be noted that most of the enzymes do derive from *B. thetaiotaomicron*.

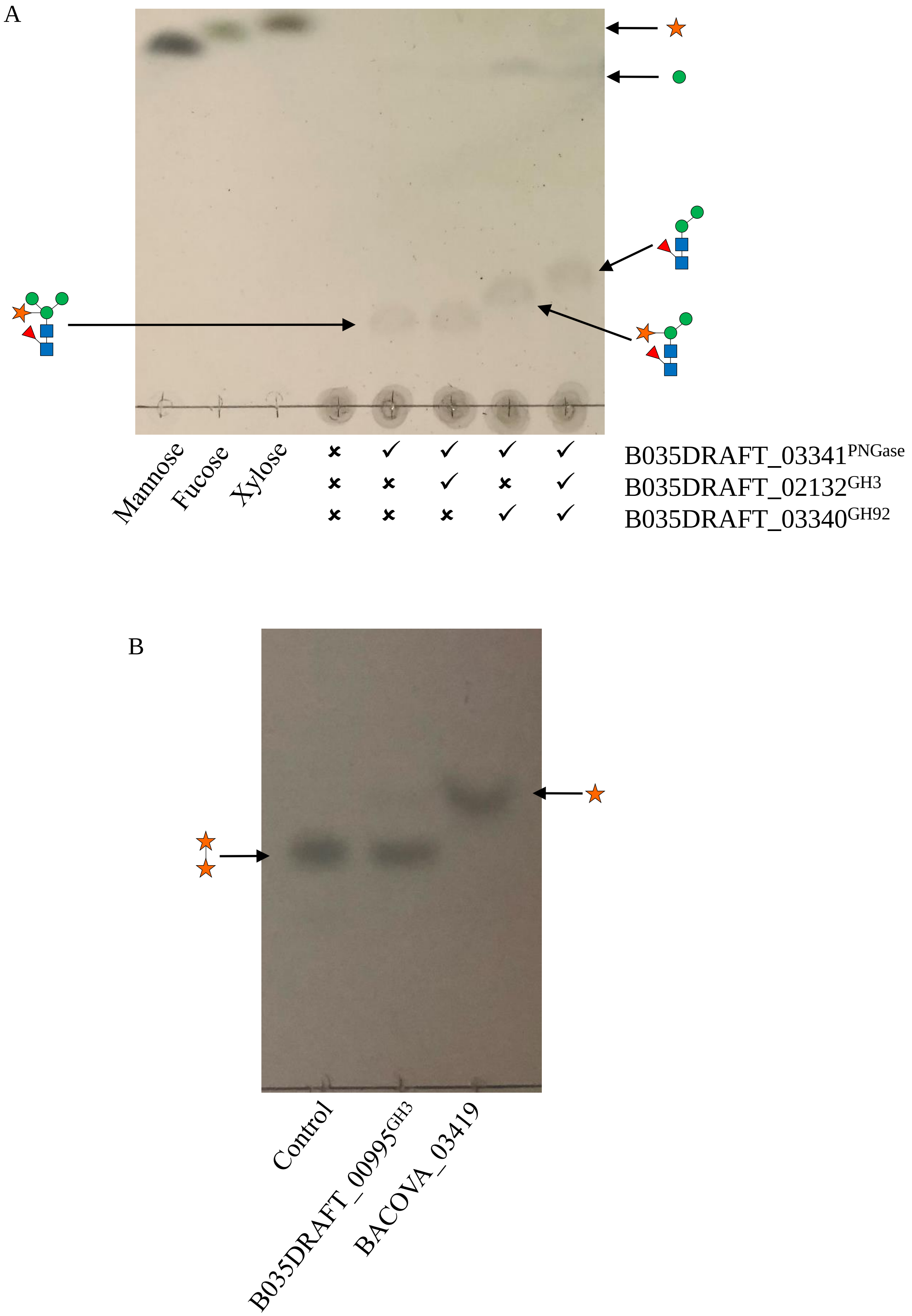

Supplementary Fig. S11. Activity of B035DRAFT_00995^{GH3} β-xylosidase. (A) B035DRAFT_00995^{GH3} was incubated against horseradish peroxidase in different combinations with B035DRAFT_03341^{PNGase} and B035DRAFT_03340^{GH92}. Assays contains 10 mg/ml of substrate 1 mM substrate, and 20 mM MOPS pH 7 and were carried out overnight at 37 °C. 9 µl of the assay in total (3x 3 µl) was spotted onto the TLC plate. The results show that B035DRAFT_00995^{GH3} cannot remove the xylose when the α-1,3-mannose is present, but once this mannose is hydrolysed by B035DRAFT_03340^{GH92} then the xylose can be removed also. (B) B035DRAFT_00995^{GH3} was incubated with β-1,4-xylobiose and compared to BACOVA_03419^{GH3}. Assays contained 1 µM enzyme, 1 mM substrate, and 20 mM MOPS pH 7 and were carried out overnight at 37 °C. 3 µl of the assay was spotted onto the TLC plate.

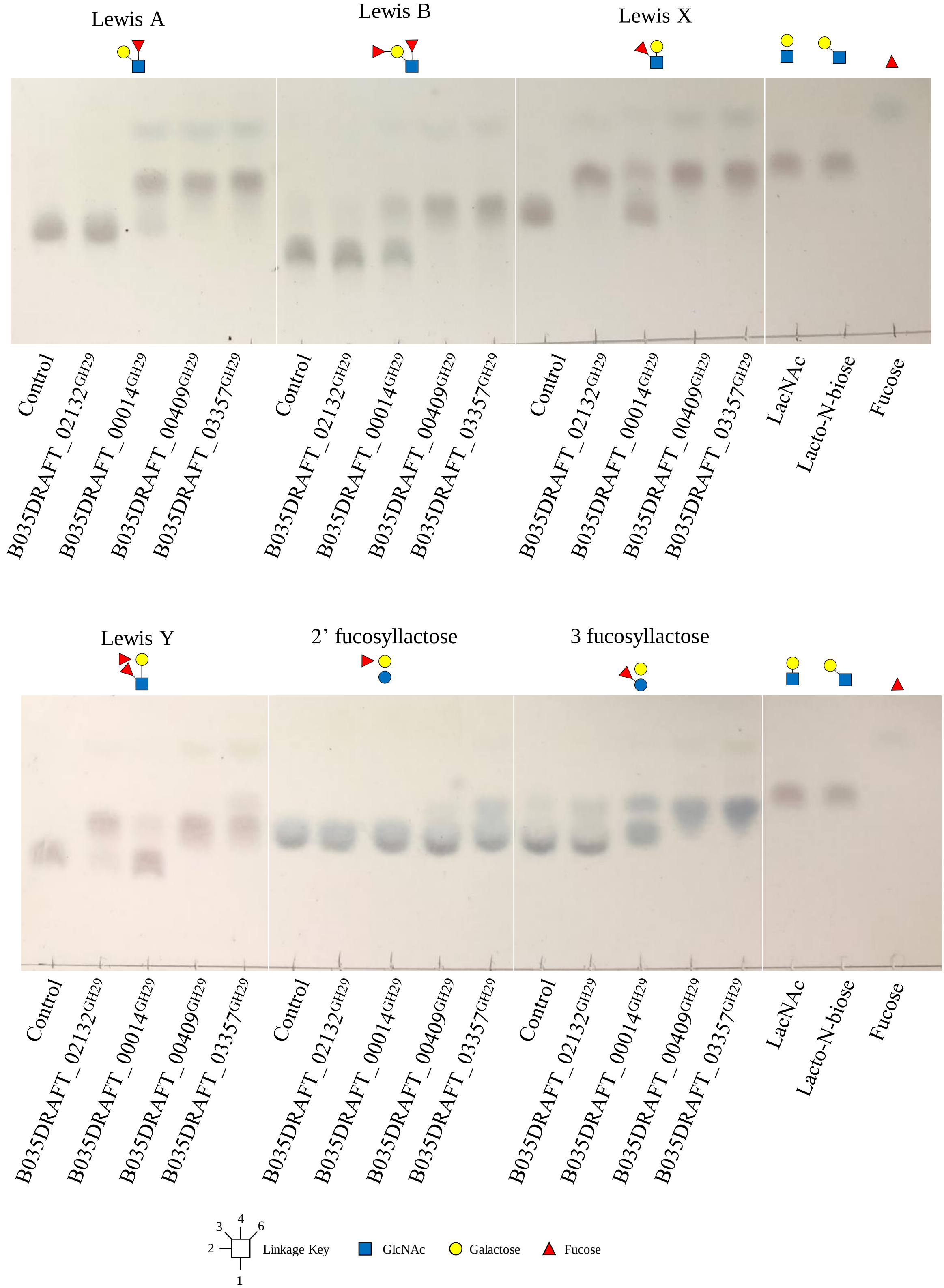

Supplementary Fig. S12. Activity of four GH29 family members from B. massiliensis. GH29 enzymes were assayed against different substrates with α-fucose decorating the non-reducing ends. Lewis A and B are the epitopes for plant and mammalian complex N-glycans, respectively. Assays contained 1 μM enzyme, 1 mM substrate, and 20 mM MOPS pH 7 and were carried out overnight at 37 °C. 3 μl of the assay was spotted onto the TLC plate.

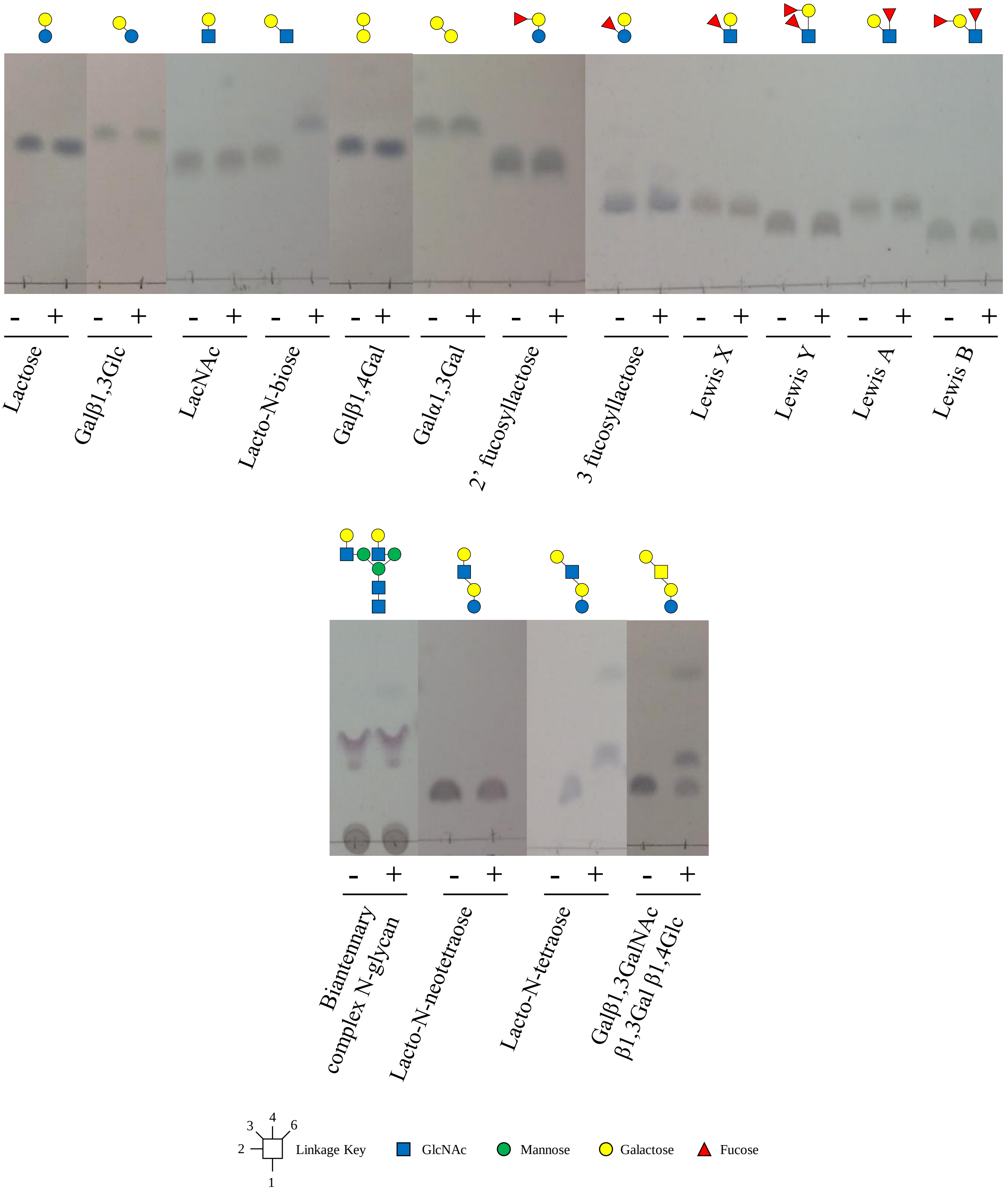

Supplementary Fig. S13. Activity of B035DRAFT_00996^{GH2} β-1,3-galactosidase. B035DRAFT_00996^{GH2} was assayed against different substrates with galactose decorating the non-reducing ends. Assays contained 1 μM enzyme, 1 mM substrate, and 20 mM MOPS pH 7 and were carried out overnight at 37 °C. 3 μl of the assay was spotted onto the TLC plate. Only the biantennary complex N-glycan was different with 10 mg/ml substrate and 9 μl of the assay was spotted onto the TLC plate.

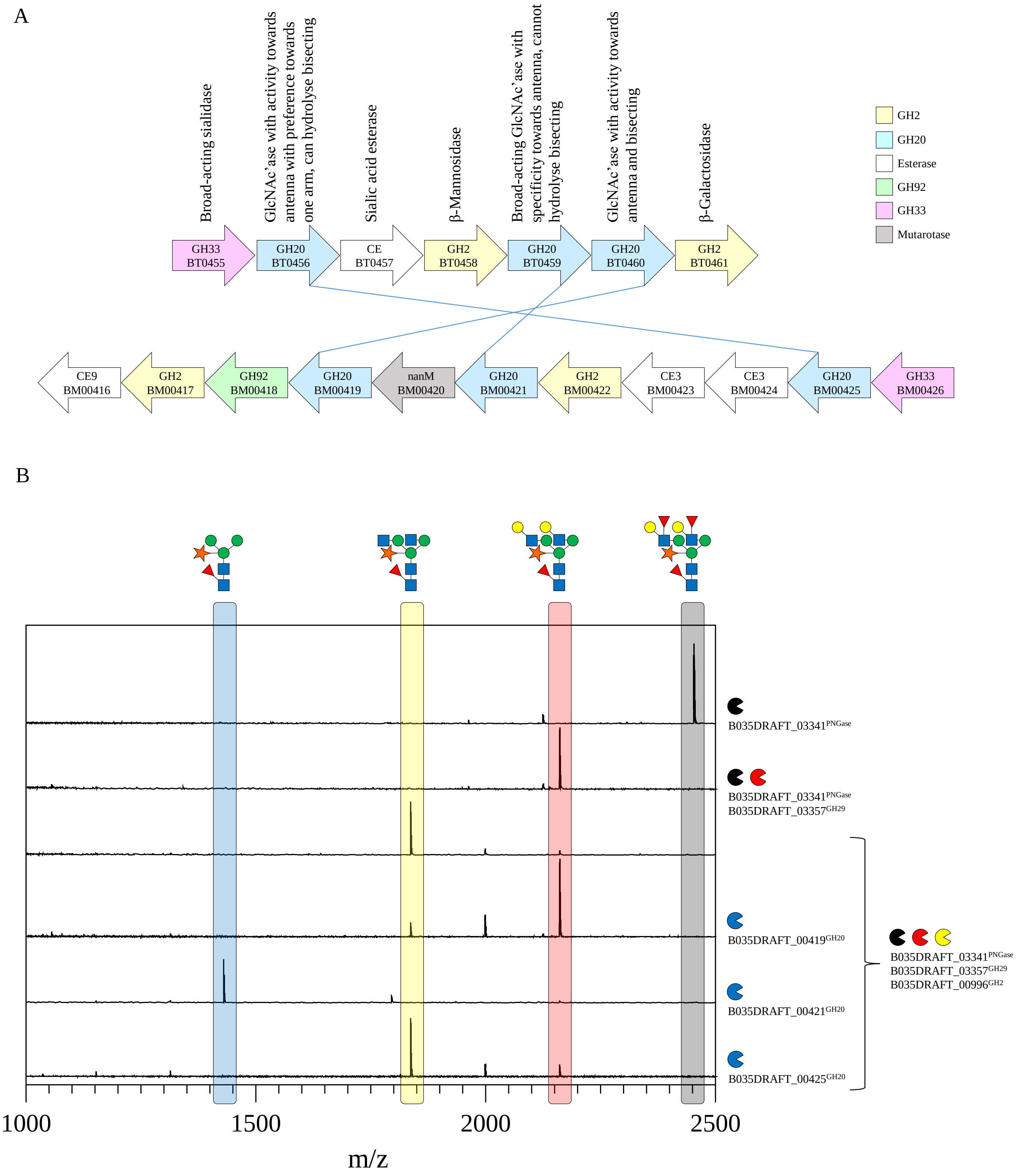

Supplementary Fig. S14. Activity of GH20 enzymes from *B. massiliensis*. (A) The previously characterised locus from *B. thetaiotaomicron* that encodes enzymes with specificity towards N-glycans and the equivalent locus in *B. massiliensis*. A summary of the enzyme activities determined for the *B. thetaiotaomicron* enzymes is provided. (B) MALDI-MS data of the GH20 enzymes tested against the antennary GlcNAc's on plant-type N-glycans. Fucosidase and galactosidase were added to each of the GH20 reactions to expose underlying GlcNAcs. The glycans were labelled with procainamide, the finished assay was spotted on to a ground steel target on top of Super-DHB matrix. Data was collected using a Bruker Auto-flex Speed in positive ion mode, range 900-3500 m/z at a 50 % laser intensity. Data was processed using Flex analysis 3.5. The data show that only B035DRAFT_00421^{GH20} can remove the antennary GlcNAc sugars.

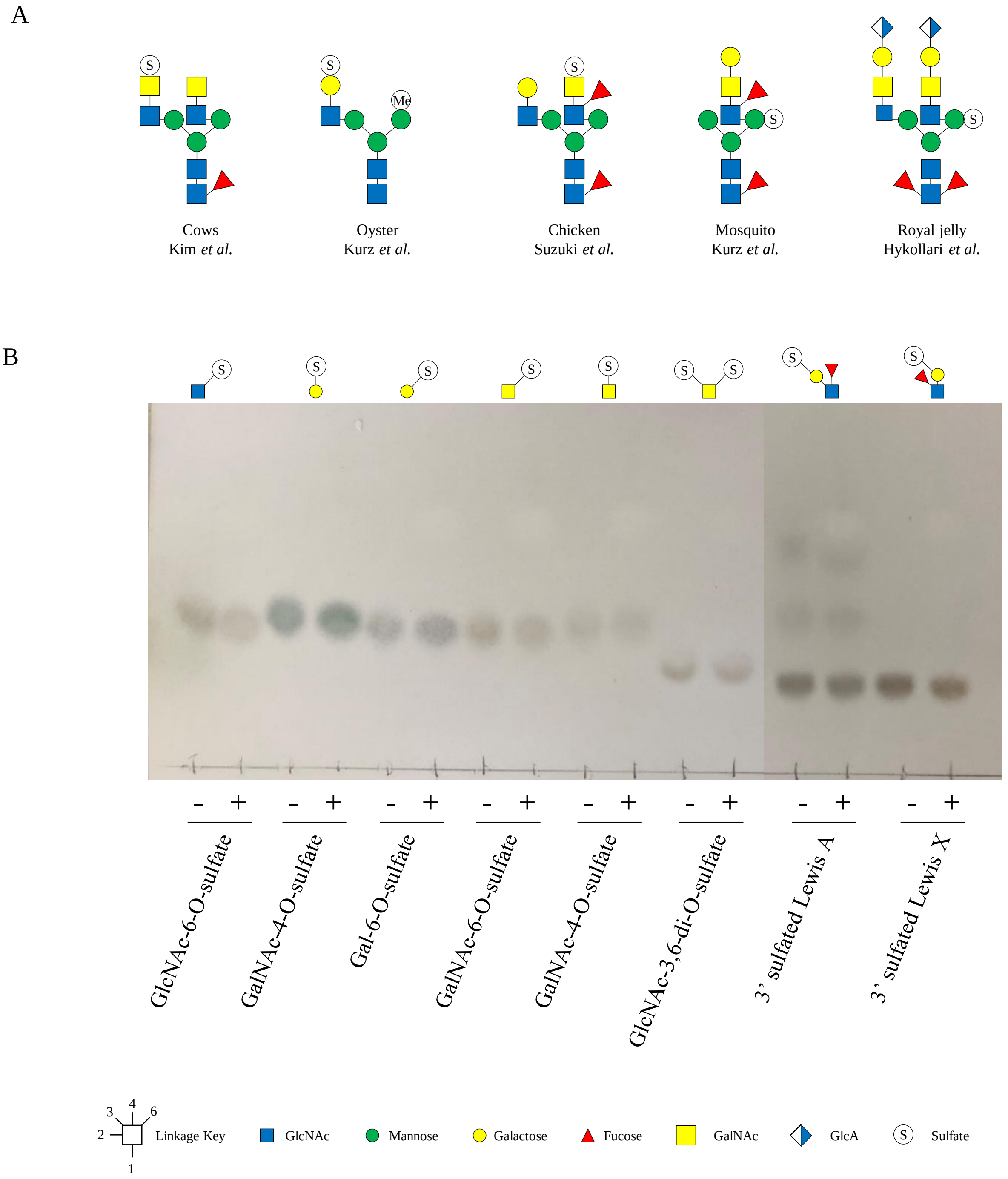

Supplementary Fig. S15. Sulfated N-glycans and testing the activity of B035DRAFT_00997^{sulfatase} (A) Structures of sulfated N-glycans that have been reported in the literature. (B) B035DRAFT_00997^{sulfatase} was assayed against different sulfated mono- and trisacchairdes. Assays contained 1 µM enzyme, 2 mM sugar, and 20 mM MOPS pH 7 and were carried out overnight at 37 °C. 3 µl of the assay was spotted onto the TLC plate.

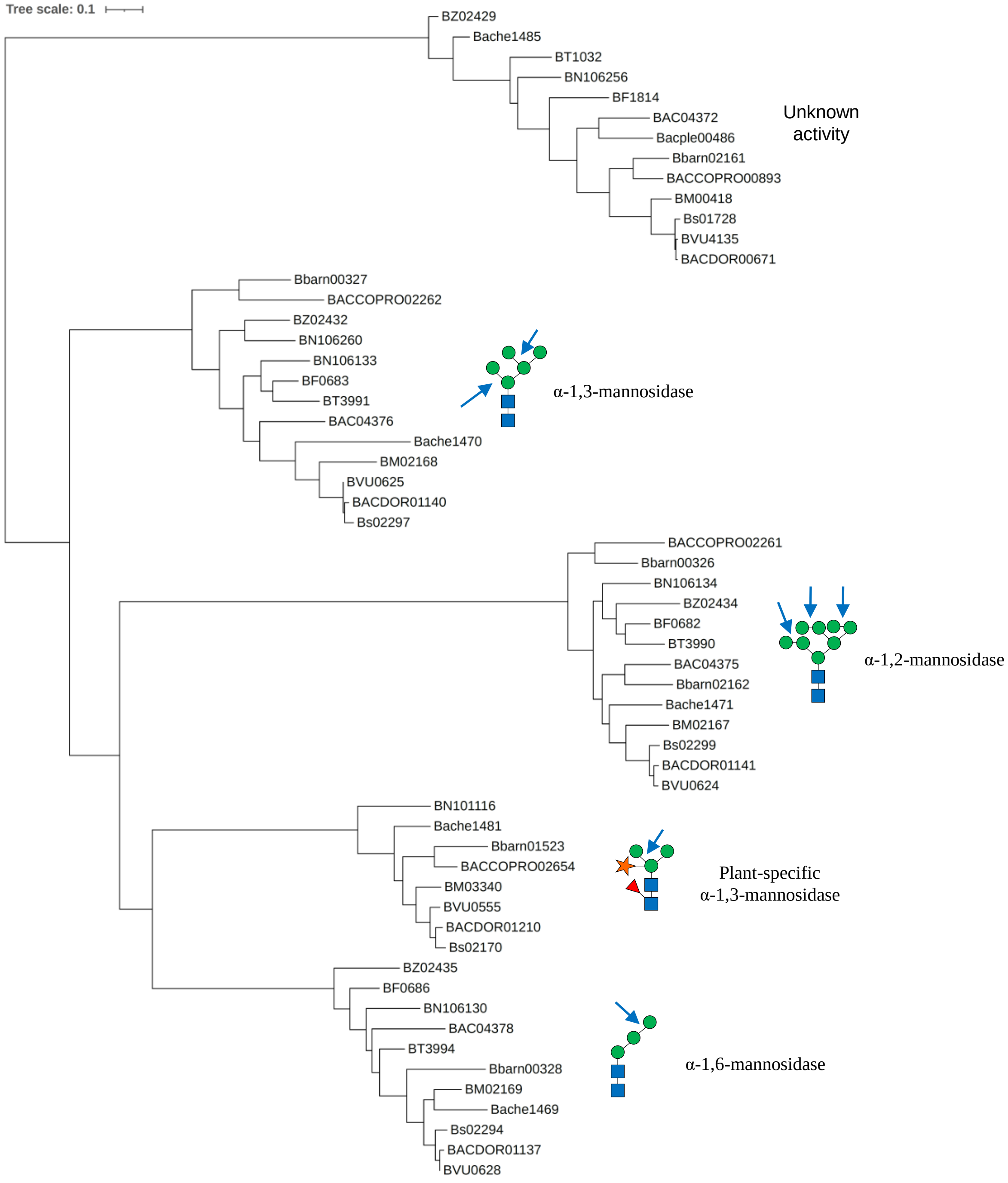

Supplementary Fig. S16. Phylogenetic tree of GH92 enzymes. Within the different groups, the GH92 enzymes with unknown function, the α-1,3-specific mannosidases, the α-1,2-specific mannosidases, the plant N-glycan α-1,3-specific mannosidases, and the α-1,6-specific mannosidases have 68-99, 61-99, 70-98, 70-96, and 69-99 % identity between members of that particular branch.
